# Statistical Viewer: a tool to upload and integrate linkage and association data as plots displayed within the Ensembl genome browser

**DOI:** 10.1186/1471-2105-6-95

**Published:** 2005-04-12

**Authors:** Judith E Stenger, Hong Xu, Carol Haynes, Elizabeth R Hauser, Margaret Pericak-Vance, Pascal J Goldschmidt-Clermont, Jeffery M Vance

**Affiliations:** 1 The Duke Center for Human Genetics, Duke University Medical Center, Durham, North Carolina 27710, USA; 2 Department of Medicine, Duke University Medical Center, Durham, NC 27710, USA

## Abstract

**Background:**

To facilitate efficient selection and the prioritization of candidate complex disease susceptibility genes for association analysis, increasingly comprehensive annotation tools are essential to integrate, visualize and analyze vast quantities of disparate data generated by genomic screens, public human genome sequence annotation and ancillary biological databases. We have developed a plug-in package for Ensembl called "Statistical Viewer" that facilitates the analysis of genomic features and annotation in the regions of interest defined by linkage analysis.

**Results:**

Statistical Viewer is an add-on package to the open-source Ensembl Genome Browser and Annotation System that displays disease study-specific linkage and/or association data as 2 dimensional plots in new panels in the context of Ensembl's Contig View and Cyto View pages. An enhanced upload server facilitates the upload of statistical data, as well as additional feature annotation to be displayed in DAS tracts, in the form of Excel Files. The Statistical View panel, drawn directly under the ideogram, illustrates lod score values for markers from a study of interest that are plotted against their position in base pairs. A module called "Get Map" easily converts the genetic locations of markers to genomic coordinates. The graph is placed under the corresponding ideogram features a synchronized vertical sliding selection box that is seamlessly integrated into Ensembl's Contig- and Cyto- View pages to choose the region to be displayed in Ensembl's "Overview" and "Detailed View" panels. To resolve Association and Fine mapping data plots, a "Detailed Statistic View" plot corresponding to the "Detailed View" may be displayed underneath.

**Conclusion:**

Features mapping to regions of linkage are accentuated when Statistic View is used in conjunction with the Distributed Annotation System (DAS) to display supplemental laboratory information such as differentially expressed disease genes in private data tracks. Statistic View is a novel and powerful visual feature that enhances Ensembl's utility as valuable resource for integrative genomic-based approaches to the identification of candidate disease susceptibility genes. At present there are no other tools that provide for the visualization of 2-dimensional plots of quantitative data scores against genomic coordinates in the context of a primary public genome annotation browser.

## Background

### The search for genes contributing to complex human diseases

The availability of the complete DNA sequence of the human genome, along with advances in gene expression, proteomics, metabolomics technology and bioinformatics databases, presents new opportunities for integrative approaches to identify candidate susceptibility genes for complex human diseases. Complex diseases, which include such diverse illnesses as Alzheimer disease, Parkinson disease, cardiovascular disease and asthma, account for the majority of chronic illnesses that plague our society today. These non-Mendelian diseases are attributable to inherited polymorphisms in perhaps several risk-associated or modifier genes that are triggered by exposure to environmental agent(s). Because of the multitude of factors ultimately contributing to the disease phenotype and the numerous confounding variables presented in studying human diseases, isolating the genetic components that confer an underlying predisposition to a complex disease is an inherently daunting undertaking.

### The importance of data integration

Thus, to improve the odds of successfully identifying complex disease susceptibility genes, several diverse approaches, each of which must capitalize on cutting-edge technical, informatics and analysis, should be exploited. The integration of disparate biological, statistical and clinical databases, both public and private, into whole-genome annotation are of paramount importance to comprehend and efficiently interpret the vast quantities of DNA sequence data, gene expression data, proteomics and other "-omics" data. As genotyping is a costly endeavor, ever-more effective computational tools are needed to readily access, organize and comprehend the massive quantities of data generated to identify and prioritize candidate genes for genotyping. Only when a disease causing genetic mutation is confirmed can the underlying molecular mechanisms of complex diseases be unraveled so that tests, prevention, new knowledge-based therapeutic approaches can eventually be devised.

Great strides have been made towards the federation of bioinformatics databases as a result of concerted efforts over the last few years by leading bioinformaticians to develop controlled vocabularies [1,2], common platforms and tools for integration [3]. As a result, despite the exponential growth of bioinformatics data, the number of individual web-based resources that genetic researchers have to navigate has been substantially reduced from a multitude of disparate web sites on single chromosomes and individual physical and genetic maps to essentially three major on-line resources that facilitate access, analysis and retrieval of data from the recently completed human genome.

The visual presentation of the immense quantities of incongruent data types, however, presents challenges in itself. Effective integrated informatics tools must be capable of representing essential data of ever increasing complexity in a format that is both comprehensive and easily synthesized by the human brain [4]. Towards this end, three well-annotated web-based public genome browsers with improved interfaces have been developed and are continually evolving. These are: 1) the NCBI Entrez map viewer [5,6], 2) the EMBL-EBI / Sanger Institute collaborative Ensembl project [7,8], and 3) the University of California at Santa Cruz's Golden Path Genome Browser [9,10]. All are easily queried and capable of visually presenting overviews of the large regions of the genome while allowing the user to zoom in on an area of interest revealing detailed information on the numerous features mapped within. Each of these has become invaluable tools in the arsenal of genetic researchers.

### Genomic convergence

We and others have embraced a multi-faceted integrated approach to identify and prioritize candidate genes for complex human diseases that we call "genomic convergence" [11]. This approach combines the list of genes obtained two or more distinctly different methods (e.g. gene expression and linkage analysis) to obtain a list of top candidate genes. Theoretically, including genes identified by other independent, yet biologically relevant, lines of evidence could increase the sensitivity of the approach as well as the specificity when the genes identified by several approaches are giving higher priority.

### Rationale

To increase the efficiency and accuracy of the identification and prioritization of candidate genes for genotyping, we needed to facilitate the identification and extraction of genomic features of interest that are within linkage regions as well as fully exploit the public databases and the human genome assembly and annotation projects. A strategy relying on the assignment somewhat arbitrary "fitness" thresholds to reduce the number of candidate genes for follow-up analysis poses the risk of excluding the causative gene from the list of candidates if the thresholds are too stringent. For this reason a tool capable of displaying quantitative and positional data within a single integrated browser view that would facilitate the synthesis and interpretation of disparate data types is superior to the more simplistic approach using the intersection of sets of genes so that the thresholds are set empirically.

### Customizing Ensembl to incorporate two-dimensional linkage and association data plots

For reasons discussed later in this paper, we opted to use a local implementation of Ensembl as the basis of our internal bioinformatics infrastructure. To fully meet our needs for integrating linkage and association data we developed software to customize Ensembl as an analytical tool for genomic convergence approaches to identify potential disease-susceptibility genes for follow-up analysis. For this purpose we have developed software modules that add functionality to the Ensembl genome annotation systems so that the browser will display quantitative data points plotted against chromosome position (e.g. statistical results, from genomic screens, fine mapping and association studies and expression levels), which are seamlessly integrated into the Contig View and Cyto View web pages. The new panels fully support the functionality of the Ensembl system so genome regions corresponding selected by the user within the Statistic View will be displayed in the Overview and Detailed View panels and additional information on the statistical data-points may be displayed in pop-up views with hyper-links to individual feature information pages. The Statistical Viewer package includes software to facilitate the upload, query, storage, integration, display, analysis, and retrieval of private quantitative data into a public open-source genome browser so that all public annotation, DAS sources and links can be fully exploited.

We have created a software package called "Statistical View" that includes an enhanced upload server that facilitates the upload, query, storage, integration, display, analysis, and retrieval of private quantitative data into a public genome browser to help geneticists make connection between the disease phenotype and the genetic features that are associated with risk on a genomic scale. From there an understanding of the molecular basis of disease can lead to testing, prevention, and perhaps ultimately, to pharmaceutical or alternative means of intervention so that the hopes of translational medicine promised by the completion of the human genome sequencing project will eventually be realized.

## Implementation

### Mapping genetic marker locations as genomic positions in the human genome assembly

Linkage data is traditionally visually displayed as a graph in which Lod scores are plotted along the ordinate against the genetic location of the markers that were used in the genetic screen and the subsequent analysis. The length of the abscissa represents the length of the chromosome in centimorgans (cM), a function of the recombination frequency between two loci. Recombination frequency is influenced by factors such as regional genetic content and gender so that one can only make a rough approximation between in the average number of base pairs that are in a single centimorgan map unit. Therefore, to incorporate a visual representation of statistical results into the Ensembl genome browser in a meaningful way, it is imperative that the abscissa be expressed in base pairs so that the position of markers along the abscissa correctly align with, and strictly correspond to, the horizontal illustration of the ideogram that is displayed immediately above the linkage study graph in the Ensembl ContigView. Towards this end we have developed a tool "GetMap" that uses linear interpolation relative to deCODE Genetics' published map [12] to approximate the physical chromosomal coordinates denoted in bps of markers that were a) not mapped by the deCODE group and b) we have insufficient information for successful ePCR. The algorithm first uses a binary search (the "divide-and-conquer" paradigm) to find and extract known marker coordinates from a database when available. Otherwise the linear interpolation algorithm is activated, using the closest markers flanking markers with known coordinates to approximate the position as accurately as possible. The algorithm is based on that described in by Kong et al. [12].

To convert the map units from centimorgans into their chromosome sequence coordinates in base pairs, it is necessary to map the markers used as probes to the physical sequence of the human genome. The most recent version of the human genome assembly (currently NCBI build 35) can be downloaded from the UCSC genome site [10] and the most recent version of the Ensembl annotation system [8].

The first step in converting the genetic location of a marker into its position in base pairs is the creation of a database of unique reliable and valid markers (typically microsatellites) based on the NCBI UniSTS database [13,14], an integrated non-redundant database of markers (sequence tagged sites, STSs [15]), as a starting point. UniSTS integrates mapping information gathered from various resources primary sources, and is the source of marker probe sequence information. The markers, their aliases, sequences and available locations on genetic maps are retrieved via the NCBI ftp server [16]. Once the most recent version of the human genome assembly and UniSTS has been uploaded onto our local servers we re-map the positions of STSs by using e-PCR [17-19], or BLAT [20] as a next resort. In a preprocessing step, markers that may have erroneous map positions and/or show inconsistent ordering when compared to other maps are flagged, removed from our verified database and saved elsewhere for reference. The confirmed genomic position and other information, including its genetic location(s), is then used to populate the statistical results table in the database illustrated Figure 3. Then, to facilitate the interconversion of map locations from pre-existing genetic maps and analysis into the human genome nucleotide position, we have developed a web-based tool called "Get Map" for uploading a file containing marker locations, as well as their position as provided by the user. This tool also exists in a stand-alone version for processing batch files and is described in the Get Map documentation file provided as supplementary material.

### Software for viewing statistical data as 2-dimensional plots within the context of Ensembl

**Figure 1:**
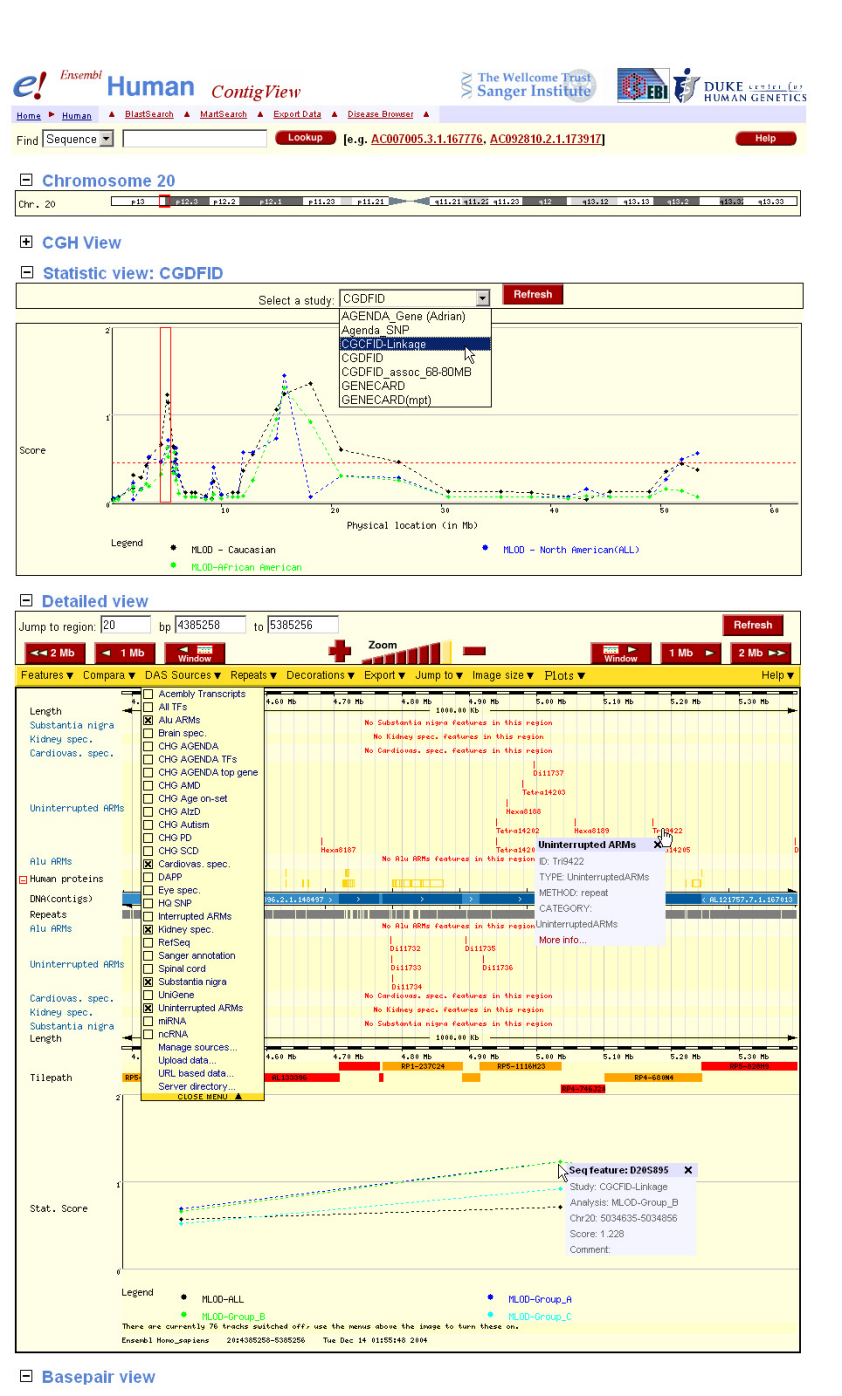
**Contig View featuring Statistic View**. Shown is a screen shot depicting a local implementation of Ensembl's contig view page hosted by the Duke Center for Human Genetics, captured using SnagIt 7.0. The figure illustrates the appearance of the Statistic View panel and some of its features in the context of Contig View. It also demonstrates how the selection of a peak can enable the researcher to easily see where linkage data and other features such as potentially unstable tri-nucleotide repeats and gene expression data converge to suggest priority gene for association and sequence analysis.

**Figure 2:**
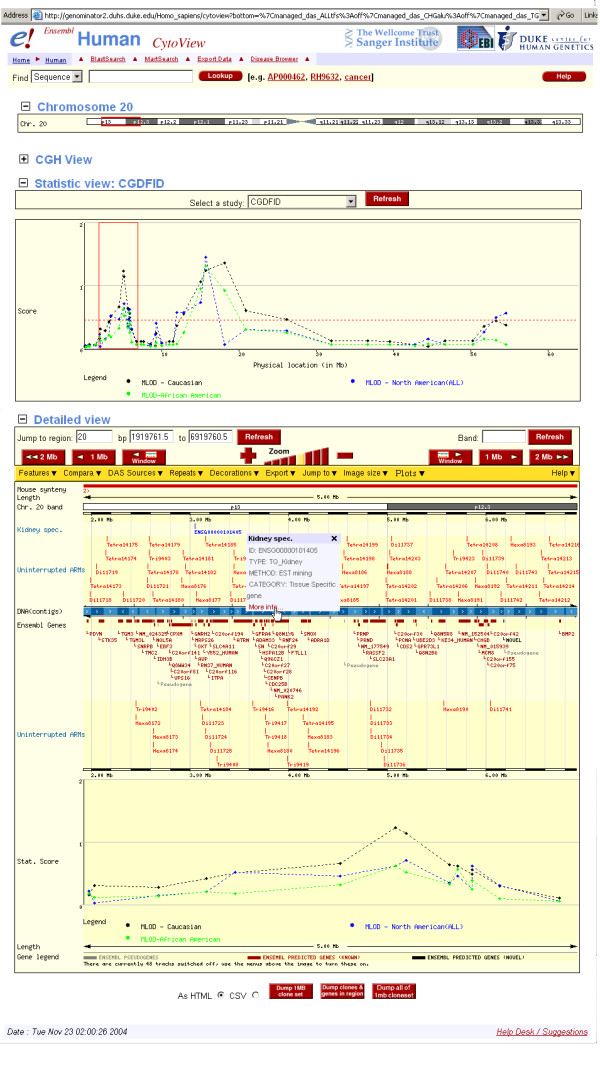
**Cyto View featuring Statistic View**. A screen shot of private data hosted at primer.duhs.duke.edu illustrating the integration of Statistic View in the Ensembl CytoView page.

The drawing program consists of four basic Bioperl modules that build on the Ensembl open source software and genome annotation system to display a linkage plot of statistical results, that are easily uploaded by laboratory personnel using the upload server we devised, in the context of the annotated human genome sequence. These modules add an additional display panel, "Statistic View" that appears automatically when the Contig View (see Figure 1) and the Detailed View in CytoView (see Figure 2) pages are opened. However, if no statistical data for any studies has been uploaded into the mySQL db for a chromosome of interest no plots are drawn, but a compressed panel appears displaying text indicating that "there is no statistical data pertaining to any study available for this chromosome" (not shown). Like other Ensembl panels, to save space, this panel can be compressed when not needed. Once the data is uploaded into a local Distributed Annotation System (DAS) [21] server, researchers can use a pull-down menu to select the plot for a particular study of interest that is then drawn in a panel placed between ideogram of the chromosome and the Overview in both Cyto View and Contig View.

### Description of BioPerl modules for embedding "Statistic View" panels in Ensembl

Using the Perl programming language we developed BioPerl modules (reviewed by Stajich et al. [22] that draw a linkage (or other) plot using the data uploaded in the DAS server for the particular study, which the researcher must provides the name of as a required field. The data flow for the Statistic View module is illustrated in figure 4. The source code is provided as supplemental material. The four basic BioPerl modules are: 1) Bio::EnsEMBL::Linkage.pm, 2) Bio::EnsEMBL::DBSQL::LinkageAdaptor.pm, 3) Bio::EnsEMBL::GlyphSet::lodplot.pm and 4) WebUserConfig::chrplot.pm and Bio::EnsEMBL::GlyphSet::FineLODplot provides for a detailed statistical plot, described below, that is drawn beneath the Detailed View panel in Ensembl's Cyto- and Contig- View pages. The source code for each of these is provided as rich text files (see Additional file 1 through 5, respectively). Also documentation and executables are provided within a compressed archive file (Additional file 6).

**Figure 3:**
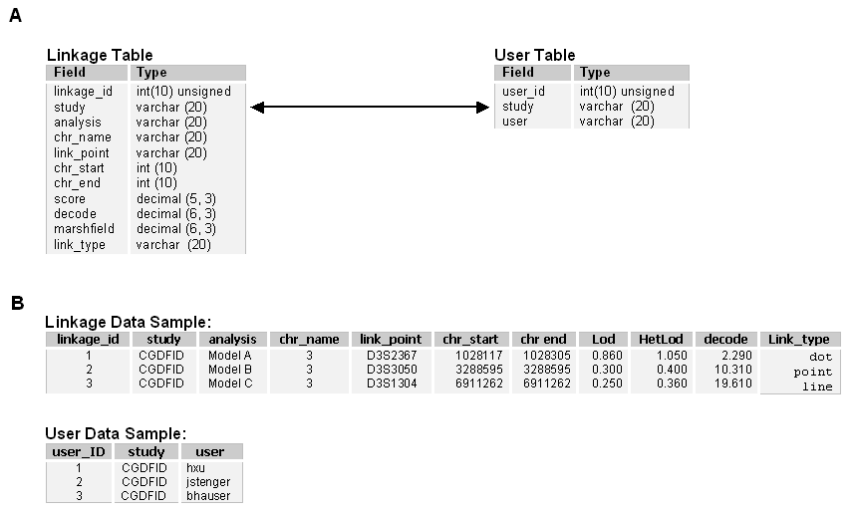
**Table structures for the linkage object and the user access database**. Part A of the figure shows that the study field serves as the key relating the User Data to the Linkage Data. Also shown are examples (part B) of three data records that define plot coordinates along with other attributes. User databases are created to allow restricted access for uploading data.

**Figure 4:**
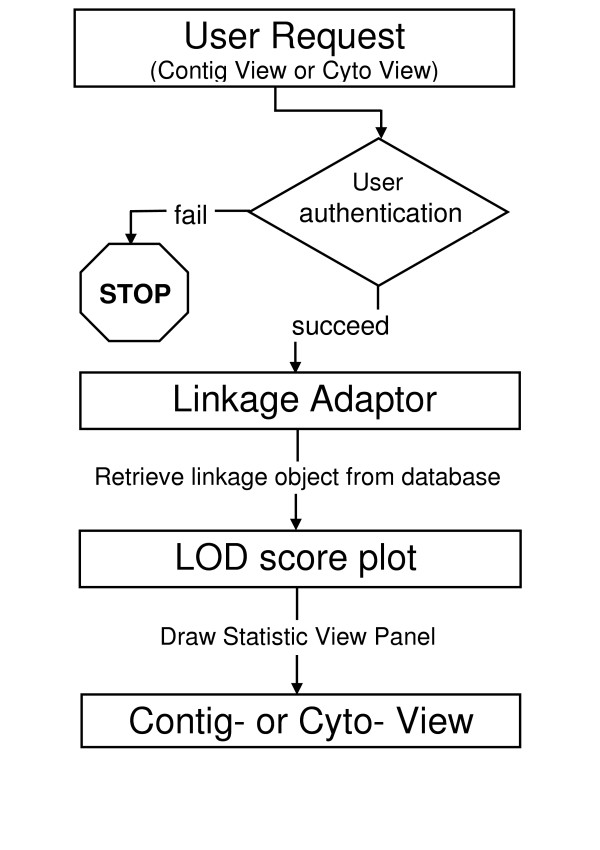
**Flow chart illustrating the flow of data in Statistic View**. A security feature is built in so that the process aborts if the user is not authenticated.

Briefly, the Linkage.pm module features a constructor to create the linkage object, essentially a table that encapsulates the record for a single linkage point, allowing for linkage data to be added populating the database. The database table, Linkage_Table, contains up to eleven fields shown in figure 3A. This information can be viewed in the Ensembl Contig- and Cyto- View browser pages within a pop-up box when moving the cursor over ("mousing-over") the point in the plot as shown in figure1 below the Detailed View)

The GlyphSet::lodplot.pm module is next implemented. This module contains all the information for drawing the graph of the statistical data. This module served as the basis for the FineLODplot.pm that displays a graph of statistical data below the Detail View Panel that corresponds to the same range (from the slice of the chromosome selected when the user slides the red box in either the ideogram or the Statistic View panel) of the Detailed View Panel. The WebUserConfig::chrplot.pm module is then added to configure the lod plot so that it is displayed in the Statistic View panel in CytoView and ContigView.

The LinkageAdaptor object module, LinkageAdaptor.pm, provides the functionality for accessing linkage data from the DAS database. Linkage adaptor creates a "slice" object, corresponding to the sliding red box that the view uses to select a region. The slice object defines the region from which linkage data will be retrieved and returns the records from the linkage objects that are mapped to the chromosome between the coordinates within the boundary of the red box that are displayed in the detailed view below. Finally the plot image was scored into the Ensembl Contig View and Cyto View by modifying the Contig- and Cyto- view code so that the linkage plot is displayed into the Ensembl Contig View.

Figure 1 is a screen shot of the Ensembl Contig View web browser running off of our local server that illustrates the Statistic View panel with a plot comparing lod and hetlod scores against their corresponding genomic location. Figure 2 similarly illustrates the incorporation of Statistic View into Ensembl's CytoView. Note how the graph in Figure 1 features data points interconnected by dotted lines while that in Figure 2 shows solid lines. The database table provides a field called link_type that allows a statistical analyst to specify the display to quickly allow one type of analysis, such as single point, from another, such as multi-point, when a convention is adhered to.

Although Statistic View is an invaluable tool for surveying chromosomes for interesting genomic features in linkage peaks derived from a genomic screen, it is of limited use in fine mapping and association studies due to insufficient resolution at the chromosomal level to see the desired features as the density of markers increases. Thus, recognizing the utility of having the ability to display detailed information in a region selected for detailed association studies, we have developed code (provided within Additional file 5) to plot and display association data in a separate panel, below the Detailed View in Contig- and Cyto- View. Essentially, this panel, that we refer to as "Detailed Statistic View" is an enlargement of a region selected from either "Statistic View" or the Overview panel that will directly corresponds with the Detailed View outlined in red (See figures 1 and 2).

## Results

Here we describe the functionality of a program package called "Statistical Viewer" that was written for the purpose of integrating statistical genetic data with human genome sequence annotation. The name "Statistical Viewer" refers to the name of the software package while "Statistic View" refers to the display panel that is labeled in Ensembl's "Contig View and Cyto View" pages.

### Usage

The first step in adding statistical data to Ensembl is formatting the data for upload. As the abscissa must correspond to genomic coordinates for integration into the human genome assembly, the position of the linkage point (for the microsatellite or other marker) needs to be defined by the name of chromosome, and chromosome start and end position in base pairs.

### Simplified data entry via an improved web-based upload service

To facilitate the upload of statistical data as well as other types of private data, we developed a customized upload server that allows members of a group with permissions to upload their own data Users may add new data to be plotted into "Statistical View" or data for annotation as DAS tracts. Our improved DAS upload server allows users to append data into previously instantiated data tracks. The web-based interface is also user-friendly so that formatting difficulties and failures and errors that typically ensue when cutting and pasting tab-delimited text into the Ensembl web form, are avoided.

### Upload file format requirements

The data must be in the form of a simple two-dimensional table containing attributes (columns) and tuples (rows). The upload serves accepts either tab-delimited text files or MS excel spreadsheet files as input using a browse feature to specify the directory. When using an Excel file, the top worksheet page must contain all the data to be uploaded and be devoid of any merged fields. We have designed the upload server to require a minimum of eight essential fields that serve as column headings in a table, although additional attributes such as the name of the researcher or technician entering the data, the method, or date are permitted and encouraged.

These eight required fields (attributes) are:

1. *Study*: The term defines the data set and is the title of the study. The field is a string of characters giving the name of a disease, project or a sub-study. One study can have data across multiple chromosomes or in several analysis groups. Data from multiple studies can be combined into a single spreadsheet if the records are listed sequentially in tuples (rows).

2. *Analysis*: This field enables representation of data sub-categories from stratification within a study. The character string can be different statistical analysis methods, or the different populations, etc. Again, several data categories can be included into the same spreadsheet. The fields will be used to provide the key or legend for the points plotted on the Statistic View graph. The units should be included in parenthesis.

3. *Link_point: *This field is typically the name of the marker for linkage study. The value will be represented as a data point on the Statistic View graph that will display the name of the marker in a pop-up window when the point is "moused-over".

4. *Score: *The statistical score for the Link_point. The units may be indicated here as well the score for inclusion in pop-up windows.

5. *Chr_name: *All human chromosome names (1 through 22 as well as X, Y) are acceptable character values.

6. *Chr_start: *The chromosome start location of the Link_point in base pairs.

7. *Chr_end: *The chromosome end location of the Link_point also in base pairs. This position may be identical to the chr_start value in the case of a SNP.

8. *Link_type: *This attribute specifies the type of line used for connecting the Link_points in the plot: for example, "dot" denotes a "dotted line (- - - - -)" that we use as a convention two-point analysis. "Line" is "solid line (____) " and typically indicates a multi-point analysis. By placing the term "point" in this field, Statistical View will draw a scatter plot without lines. If a value is not supplied for this field a solid line, the default, will connect the link points.

### Viewing integrated statical data in Ensembl

The StatisticalView panel depicts a graph plotting the linkage, or association, statistics along the ordinate and the length of the chromosome, in megabases (Mbs), along the abscissa. The length of the abscissa diametrically corresponds to the length of the ideogram that is illustrated directly above. The program generates this panel, which is capable of displaying two-dimensional graphs plotting data for pertaining to a study of interest. Refreshing the page following selection of another study or analysis method can bring up different plots. This plot is seamlessly integrated into the Cyto View and the Contig View pages. Like other panels in these web pages, the Statistic View panel features a selection box, a movable red rectangle with adjustable width, to highlight the boundaries of a region of interest in the linkage plot. The boundaries of the region selected in Statistic View directly correspond to the selection box in the ideogram and the physical map position that is denoted in bps in the Detailed View. The selection box also correctly corresponds with the enlarged box that is dynamically drawn in the overview. As with the corresponding selection box in Ensembl's other display panels in Contig- and Cyto- View, the width of the selection box in Statistic View can be altered with a mouse, or a similar input devise, to select either a larger or a narrower region of interest to dynamically alter the other display panels. Likewise, changing bp coordinates in the Detailed View, or altering the size or location of any of the sliding selection boxes in the other panels, has the appropriate effect on the selection box in the Statistic View panel.

## Discussion

Following the much-anticipated first release of the draft sequence of the human genome by the international consortium [23] and the Celera Corporation [24] in February 2001, human geneticists were eager to apply this resource data to use to map and identify disease susceptibility genes. Even in its incomplete and unverified state, the data represented a tremendously powerful resource to help resolve the inconsistencies that confounded the use of independently derived genetic and radiation hybrid maps. Because of the computational complexity of dealing with large and incomplete human pedigrees [25], the production of these maps was a significant accomplishment. The deCODE Corporation immediately incorporated the draft sequence data in constructing a new meiotic map [12] that represented a significant improvement over the Marshfield [26] and Genethon maps [27]. It was also immediately obvious that a tremendous amount of work remains to translate this data into knowledge that will eventually improve the overall health of the public and that the processes of analyzing and interpreting this data presents many challenges in itself.

In spite of the exponential growth of biomedical data, the task of mining data is less daunting than it was just a few years ago. Via the World Wide Web (WWW) geneticists have at their disposal three distinct, high-quality, well-annotated genome repositories that provide free access to the most recent genome assemblies for humans as well as an increasing, diverse assortment of model organisms. Each of these public genome browsers, NCBI, UCSC and EBI/Sanger's Ensembl, employ their own annotation pipeline but should contain the same nucleotide sequence from the latest, or at least the second most recent, release of the NCBI assembly. As is the case with the NCBI and UCSC genome browsers, the public Ensembl site contains genomic sequences and a plethora of useful features extending well beyond known and predicted genes, microsatellite markers, and SNPs that are linked to their corresponding records hosted at their respective primary sources (see [28] for a review of current molecular biology databases).

We have chosen Ensembl as the system to underlie Statistic View for several reasons: 1) the horizontal presentation of the genome annotation makes it amenable to displaying a linkage map, 2) Ensembl has stably incorporated DAS for displaying customized data from sources outside of the Ensembl annotation pipeline mapped to the genome as tracts for several years, 3) the developers have intended from the start that the project would be open source and thus have taken great care in documenting its source code, and 4) Ensembl's EnsMart genome data retrieval tool is a very sophisticated and flexible data mining tool containing extensive filters and several good output options. Thus for the purpose of integrating linkage data, as well as other types of internal data, we believe Ensembl currently provides the best architecture to serve as the basis of an data integration infrastructure for use by a genetics laboratory in an academic setting.

A local implementation of the Ensembl genome annotation databases and software system is ideal for integrating third party annotation features with public human genome sequence annotation and serves as the foundation of our bioinformatics infrastructure to assist in the identification and prioritization of candidate genes in disease fine mapping and association studies. The DAS system has enabled us to display the location of markers used in a particular study as features in tracks displayed in Contig- and Cyto-View. Additionally we have mapped other features such as the location of differentially expressed genes from experiments using both microarray [29] and SAGE technology [11]. These studies employed Statistic View and illustrate the utility of having such a tool for rapidly identifying and visualizing genomic features that are in regions of linkage.

We have found that the benefits of locally maintaining a mirror of the public human genome sequence along with the Ensembl genome browser software and a DAS server exceed the costs. It was far more costly to maintain individual databases with different formats across different projects. This integrated system allows users to manually curate genomic, computationally derived, statistical, genetic, and experimental (e.g. gene expression) results for many projects. Additionally, sensitive data can be password protected.

Although the ability to map the location of microsatellite or SNP markers used in genomic screens and genotyping into DAS tracts in the Ensembl overview has proved to be very useful in genomic convergence or integrative genomic strategies thus far, Statistic View will continue to enable us to efficiently screen regions of the genome for additional features that may be indicative of a gene warranting further investigation. Towards this end, we are conducting research to identifier better predictors of successful outcome. We have mapped to the genome sequence DNA motifs that we predict are "at-risk" for genome instability such as full length, highly identical, closely spaced inverted repeats such as Alu pairs [30,31] and long simple tandem repeats (Stenger, unpublished) in coding sequences (see detailed view in Figures 1 and 2). Displaying these features in DAS tracts in conjunction with linkage data has enabled us to hone in on a potential candidate gene even when the function of gene is unknown. Although the number of genes of unknown function is dwindling with better recognition of pseudogenes, they still represent a large enough portion of genome that it is prudent to not exclude from follow-up sequencing and association studies. However, genes of unknown function frequently are overlooked since such genes are excluded from strategies based on biological plausibility and may not be represented on microarrays.

In addition, we are in the process of mapping *trans*-acting transcription factors to the genome. Aberrantly expressed genes may not map to linkage regions, but it is likely that co-expressed genes are regulated by a common *cis*-acting regulatory element. The identification of proteins that may bind to these regulatory motifs may further inform our search for candidate genes in regions displayed in Statistic View. We have also been using *in silico *subtractive hybridization methods to identify genes expressed uniquely in tissues that exhibit pathology in the diseases that are under investigation (e.g. a DAS tract displaying the location of genes specific to the *substantia nigra *may help identify candidate genes for Parkinson Disease) and have mapped these to the genome as DAS tracks.

It is worthwhile to mention that the upload of data into the DAS database using Ensembl's upload server can be problematic. The upload server provided on the public server had a number of shortcomings: 1) it is often difficult to properly format the data as text so that the desired output is generated, 2) a full email address is required for the user ID at login, and 3) each login results in data put into a separate track so that individual records could not easily be added to an existing data track. To overcome this, the user who initialized the track was required to delete the original data file and append the new data to a file and reload the entire data set. These limitations detracted from the usefulness of our private Ensembl-DAS system because data entry became a bottleneck in the analysis project with bioinformaticians serving as gatekeepers. We extended the capabilities of the upload server so that researchers with appropriate permissions could easily add individual records to the DAS database without having to delete and recreate a new data set and its resulting tract displayed in the detailed view panel. By improving the data upload procedure, laboratory personnel who generate data can enter and maintain their data with minimal training.

To facilitate the upload of data, we developed a utility that thus far represents significant improvements over the upload server provided with the Ensembl package. Our tool works well for uploading data with base pair coordinates that is to be displayed in tracks in Contig- and Cyto- View's Detailed View panel. We have found that to have data entry proceed at the same rate as data generation, it is best to provide laboratory researchers with the tools to add their own annotation when high throughput genotyping methods are being used, although some labs may prefer to have a gatekeeper to manually curate data at the time of entry to ensure that data integrity is preserved. Recognizing the need to minimize errors, which are inevitably propagated, and faced with limited human resources to enter and curate data we in the process of developing greater functionality to our upload server by automating conversion to the proper physical location so that potential mapping errors are avoided. This software is provided in its current level of development as Additional files 7 and 8and their respective user manuals are provided in Additional files 9 and 10.

## Conclusion

We have developed software to enhance the Ensembl open source software package as a private laboratory bioinformatics infrastructure to assist in the identification of candidate complex human disease susceptibility genes. We have improved the upload server thereby empowering laboratory personnel to add project specific data to the local DAS database for display in the context of the human genome using the Ensembl genome browser's contig view. By creating an additional panel in Ensembl's Contig View and Cyto View, called Statistic View, we are able to display statistical results from gene mapping experiments in the context of human genome sequence annotation. Statistic View displays a plot of linkage and association statistics directly above the Overview. Statistic View is fully and seamlessly integrated into the Ensembl genome browser. The user can navigate the chromosome by mousing-over a selection box outlined in red that directly corresponds to the one drawn by the Ensembl software in the ideogram of the chromosome. This capability facilitates the selection of regions of the chromosome in linkage disequilibrium for easily visualization of features mapped therein that are displayed in data tracts below in the Overview. This capability allows rapid screening of regions of interest to a particular study to identify genes that deserve further screening.

## Availability and requirements

The source code is provided as supplementary material for this publication and will also be available from the Duke Center for Human Genetics public web pages [32] as well as through the public Ensembl site [8].

### Setting up a local implementation of Ensembl

To use this software the Ensembl Genome Browser Software Annotation System must be installed and running locally. The Statistical View package runs on version 26.1, which can be accessed through the Download Ensembl Wiki web URL [33]. Genome files and other database files can be access through the Ensembl ftp server [34]. Ensembl requires a server with a UNIX or Linux type of operating system (e.g. OS X, SGI's IRIX and Sun's Solaris).

All software and the full complement of mySQL genome sequences and databases currently occupies 150 gigabytes of storage space and requires just as much swap space, but may not be needed depending on your requirements as specified in the installation pdf file [35].

We maintain a current local implementation of the Ensembl open source software system, including the human genome sequence assembly and related databases running on a SunFire 12 K reference server in conjunction with a separate server, a Sun Blade 2000 acting as an annotation server to store and overlay public genome data with private laboratory data for integration using the Distributed Annotation System.

## List of abbreviations

BLAT – BLAST-Like Alignment Tool

cM – centimorgan, a single map unit

db – database

DAS – Distributed Annotation System

DNA – deoxyribonucleic acid

EMBLI – European Molecular Biology Laboratory

EBI – European Bioinformatics Institute

Mb – megabase, one million base pairs

NCBI – National Center for Biotechnology Information

SAGE – Serial Analysis of Gene Expression

SNP – single nucleotide polymorphism

SQL – structured query language

STS – sequence tagged sites (markers)

## Authors' contributions

**J E S **– scientific director of CHG bioinformatics core, headed bioinformatics database integration project, contributed to concept, implementation ideas, helped formulate requirements and provided suggestions for improvements. Secured funding for hardware.

**H X **– senior programmer responsible for implementation.

**C H** – member of database integration and post-processing groups, contributed to concept, and implementation ideas. Helped design database tables, helped formulate requirements and provided suggestions for improvements.

**E R H **– Principal Investigator for CEGS bioinformatics Component, headed post-processing project, contributed to concept, implementation ideas and requirements

**M P-V **– Director of CHG, provided funding, human resources and the impetus for the project

**P J G-C **– Chairman of the Duke Department of Medicine, provided funding and supported project development.

**J M V **– senior author, provided cause for action, contributed ideas and requirements and funding

## Supplementary Material

Additional File 1 The source code for Bio::EnsEMBL::DBSQL::LinkageAdaptor Click here for file

Additional File 6 The executable BioPerl modules and documentation can be decompressed with either the GNU (Free Software Foundation) unzip command [36] in UNIX or the WinZip Windows archive utility (WinZip Computing, Inc.) [37] in an MS Windows OS. Please see the respective web sites that are referenced for additional details on acquiring and using these compression utilities, as well as the UNIX man pages for the *tar *command: **i. Readme **A text file the provides a description of modules and the path to the code in the Ensembl Server Root directory **ii. Support.pm **(Format: Perl Module) Modified source code for the ContigView::Support package that includes the Duke Center for Human Genetics extension to add the LOD score plot panel **iii. Chrplot.pm **(Format: Perl Module) The WebUserConfig::chrplot BioPerl module **iv. Contigview **(Format: text file) Modified source code for the configuration based version of the Ensembl Contig view package including the necessary additions to incorporate the LOD score or other statistical data plot **v. Cytoview **(Format: text file) Source code for the Ensembl Cyto view package including the modifications to incorporate the LOD score plot **vi. DBAdaptor.pm **(Format: Perl Module) The modified source code for the Bio::EnsEMBL::DBSQL::DBAdaptor module that includes the Duke Center for Human Genetics extension to add the LOD score plot panel into the genome browser **vii. Linkage.sql **(Format: Structured Query Language) This file specifies the table structure for the linkage object **viii. Linkage.pm **(Format: Perl Module) Bio::EnsEMBL::Linkage Ensembl BioPerl module **ix. LinkageAdaptor.pm **(Format: Perl Module) Bio::EnsEMBL::DBSQL::LinkageAdaptor BioPerl module This Module includes the POD documentation – the main documents preceding the code **x. Linkageview **(Format: BioPerl package) This file provides the package to display the chromosome linkage plot and the information for each linkage point **xi. Linkageview.pm **(Format: Perl Module) The EnsEMBL::Web::UserConfig::linkageview module **xii. Lodplot.pm **(Format: Perl Module) The Bio::EnsEMBL::GlyphSet::LodPlot module **xiii. HTML.pm **(Format: Perl Module) This file is the modified source code for the ContigView::HTML package that includes the Duke Center for Human Genetics extension to add the LOD score plot panel Click here for file

Additional File 5 The source code for Bio::EnsEMBL::GlyphSet::FineLODplot BioPerl module Click here for file

Additional File 7 The source code for the CHG enhanced upload service for integrating local data as features for display in the Ensembl genome browser as DAS tracks Click here for file

Additional File 8 The source code for the CHG enhanced upload service for integrating linkage or other statistical data for display in plots (graphs) as a panel within the Ensembl genome browser Contig- and Cyto- View pages. Click here for file

Additional File 9 User manual for uploading Linkage or other statistical data to be plotted in a display panel called "Statistic View" within the Contig- and Cyto- view genome Browser pages of a local implementation of Ensembl. Click here for file

Additional File 10 The manual for using the Duke CHG enhanced upload server web interface to import data into MySQL database so that the features can be displayed as DAS-tracks showing annotation for experimental data within the context of the genome sequence assembly on a local implementation of Ensembl. Click here for file

Additional File 2 The source code for Bio::EnsEMBL::Linkage Click here for file

Additional File 3 The source code for Bio::EnsEMBL::GlyphSet::LodPlot Click here for file

Additional File 4 The source code for WebUserConfig::chrplot BioPerl module Click here for file

Additional File 11 Manual for using the Get Map tool for the interconversion of genomic and genetic positions for markers. The algorithm is also described. Click here for file
