# Supplementary material for: Statistical Viewer: a tool to upload and integrate linkage and association data as plots displayed within the Ensembl genome browser
Source: BMC Bioinformatics. 2005 Apr 12;6:95. doi: 10.1186/1471-2105-6-95 (PMC1087836; doi:10.1186/1471-2105-6-95)
Supplement: Additional File 1 — The source code for Bio::EnsEMBL::DBSQL::LinkageAdaptor [file 1471-2105-6-95-S1.rtf]

######################################################################
#                                                                    #
# Ensembl module for Bio::EnsEMBL::DBSQL::LinkageAdaptor             #
#                                                                    #
# Maintained by Hong Xu <hxu@chg.duhs.duke.edu>				   # 
# Center for Human Genetics Bioinformatics Core          		   #
# Duke University Medical Center                                     #
#                                                                    #
#                                                                    #
# You may distribute this module under the same terms as perl itself #
#  											   #
# POD documentation - main docs before the code                      #
#                                                                    #
######################################################################


# POD documentation - main docs before the code

=head1 NAME

Bio::EnsEMBL::DBSQL::LinkageAdaptor

=head1 SYNOPSIS

$link_adaptor = $db_adaptor->get_LinkageAdaptor();
foreach $link ( $link_adaptor->fetch_all_by_chr_name($chr) ) {
  #do something with link
}

=head1 DESCRIPTION

Database adaptor to provide access to Linkage objects

=head1 AUTHOR

Hong Xu

This modules is part of the Ensembl project http://www.ensembl.org

=head1 CONTACT

Email hongxu@duke.edu

=head1 APPENDIX

The rest of the documentation details each of the object methods. Internal 
methods are usually preceded with a _

=cut


# Let the code begin...


package Bio::EnsEMBL::DBSQL::LinkageAdaptor;
use vars qw(@ISA);
use Bio::EnsEMBL::Linkage;
use strict;

# Object preamble - inherits from Bio::EnsEMBL::BaseAdaptor
use Bio::EnsEMBL::DBSQL::BaseAdaptor;
@ISA = qw(Bio::EnsEMBL::DBSQL::BaseAdaptor);


# inherit new from BaseAdaptor


=head2 fetch_all_by_chr_name

  Arg [1]    : string $chr_name
               Name of the chromosome from which to retrieve linkage objects 
  Example    : @links=@{$linkage_adaptor->fetch_all_by_chr_name('X')}; 
  Description: Fetches all the linkage objects from the database for the
               given chromosome. 
  Returntype : listref of Bio::EnsEMBL::Linkage in chromosomal 
               (assembly) coordinates 
  Exceptions : none 
  Caller     : general 

=cut

sub fetch_all_by_chr_name {
    my ($self,$chr_name) = @_;

    my $sth = $self->prepare(
        "SELECT study, analysis, link_point, chr_start, chr_end, score
         FROM linkage 
         WHERE chr_name = ? 
ORDER BY study, analysis, chr_start"
    );
    $sth->execute( $chr_name );
    
    my ($study,$analysis,$link_point,$chr_start,$chr_end,$score) = ();
    my @out = ();

    while(($study,$analysis,$link_point,$chr_start,$chr_end,$score) = $sth->fetchrow_array()) {
      last unless defined $chr_start;
      
      my $link_obj = Bio::EnsEMBL::Linkage->new();
      $link_obj->study($study);
      $link_obj->analysis($analysis);
      $link_obj->chr_name($chr_name);
      $link_obj->link_point($link_point);
      $link_obj->start($chr_start);
      $link_obj->end($chr_end);
      $link_obj->score($score);
      
      push @out, $link_obj;
    }
    return \@out;
}


=head2 fetch_all_by_Slice

  Arg [1]    : Bio::EnsEMBL::Slice $slice
               The slice object covering the region to retrieve linkages from 
  Example    : @links = @{$linkage_adaptor->fetch_all_by_Slice($slice)};
  Description: Fetches linkage object from the database for the
               region given by the slice.
  Returntype : listref of Bio::EnsEMBL::Linkage objects in 
               Slice coordinates
  Exceptions : none
  Caller     : Bio::EnsEMBL::Slice::get_Linkages 

=cut

sub fetch_all_by_Slice {
  my ($self,$slice) = @_;
  
  my $start = $slice->chr_start();
  my $end = $slice->chr_end();
  my $chr_name = $slice->chr_name();

  my $sth = $self->prepare(" SELECT study, analysis, link_point, chr_start, chr_end, score
FROM linkage 
WHERE chr_name = ?
AND $start <= chr_end 
AND $end > chr_start 
ORDER BY study, analysis, chr_start
    ");

  $sth->execute($chr_name);

   my ($study,$analysis,$link_point,$chr_start,$chr_end,$score) = ();
   my @out = ();

   while(($study,$analysis,$link_point,$chr_start,$chr_end,$score) = $sth->fetchrow_array()) {
     last unless defined $chr_start;

     my $link_obj = Bio::EnsEMBL::Linkage->new();
     $link_obj->study($study);
     $link_obj->analysis($analysis);
     $link_obj->chr_name($chr_name);
     $link_obj->link_point($link_point);
     #convert to slice coordinates
     $link_obj->start($chr_start - $start + 1);
     $link_obj->end($chr_end - $start + 1);
     $link_obj->score($score);

     push @out, $link_obj;
  }
  return \@out;
}


=head2 fetch_all_by_study

  Arg [1]    : string $study
               Name of the study from which to retrieve linkage objects 
  Example    : @links=@{$linkage_adaptor->fetch_all_by_study('PD')}; 
  Description: Fetches all the linkage objects from the database for the
               given study. 
  Returntype : listref of Bio::EnsEMBL::Linkage in chromosomal 
               (assembly) coordinates 
  Exceptions : none 
  Caller     : general 

=cut

sub fetch_all_by_study {
    my ($self,$istudy) = @_;

    my $sth = $self->prepare(
        "SELECT study, analysis,chr_name, link_point, chr_start, chr_end, score
         FROM linkage 
         WHERE study = ?
ORDER BY analysis, chr_name, chr_start"
    );
    $sth->execute( $istudy );
    
    my ($study,$analysis,$chr_name,$link_point,$chr_start,$chr_end,$score) = ();
    my @out = ();

    while(($study,$analysis,$chr_name,$link_point,$chr_start,$chr_end,$score) = $sth->fetchrow_array()) {
      last unless defined $chr_start;
      
      my $link_obj = Bio::EnsEMBL::Linkage->new();
      $link_obj->study($study);
      $link_obj->analysis($analysis);
      $link_obj->chr_name($chr_name);
      $link_obj->link_point($link_point);
      $link_obj->start($chr_start);
      $link_obj->end($chr_end);
      $link_obj->score($score);
      
      push @out, $link_obj;
    }
    return \@out;
}


=head2 fetch_all_by_link_point

  Arg [1]    : string $study
               Name of the study from which to retrieve linkage objects 
  Arg [2]    : string $link
               Name of the linkage point 
  Example    : @links=@{$linkage_adaptor->fetch_all_by_link_point('PD', 'D1S1145')}; 
  Description: Fetches all the linkage objects from the database for the
               given study and linkage point. 
  Returntype : listref of Bio::EnsEMBL::Linkage in chromosomal 
               (assembly) coordinates 
  Exceptions : none 
  Caller     : general 

=cut

sub fetch_all_by_link_point {
    my ($self,$istudy,$link_pnt) = @_;

    my $sth = $self->prepare(
        "SELECT study, analysis,chr_name, link_point, chr_start, chr_end, score
         FROM linkage 
         WHERE study = ? and link_point = ?
ORDER BY analysis, chr_name, chr_start"
    );
    $sth->execute( $istudy, $link_pnt );
    
    my ($study,$analysis,$chr_name,$link_point,$chr_start,$chr_end,$score) = ();
    my @out = ();

    while(($study,$analysis,$chr_name,$link_point,$chr_start,$chr_end,$score) = $sth->fetchrow_array()) {
      last unless defined $chr_start;
      
      my $link_obj = Bio::EnsEMBL::Linkage->new();
      $link_obj->study($study);
      $link_obj->analysis($analysis);
      $link_obj->chr_name($chr_name);
      $link_obj->link_point($link_point);
      $link_obj->start($chr_start);
      $link_obj->end($chr_end);
      $link_obj->score($score);
      
      push @out, $link_obj;
    }
    return \@out;
}


=head2 fetch_all_by_study_chr

  Arg [1]    : string $study
               Name of the study from which to retrieve linkage objects 
  Arg [2]    : string $chr
               Name of the chromosome from which to retrieve linkage objects
  Example    : @links=@{$linkage_adaptor->fetch_all_by_link_point('PD', '15')}; 
  Description: Fetches all the linkage objects from the database for the
               given study and chromosome. 
  Returntype : listref of Bio::EnsEMBL::Linkage in chromosomal 
               (assembly) coordinates 
  Exceptions : none 
  Caller     : general 

=cut

sub fetch_all_by_study_chr {
    my ($self,$istudy,$chr) = @_;

    my $sth = $self->prepare(
        "SELECT study, analysis, chr_name, link_point, chr_start, chr_end, score
         FROM linkage 
         WHERE study = ? and chr_name = ?
ORDER BY analysis, chr_start"
    );
    $sth->execute( $istudy, $chr );
    
    my ($study,$analysis,$chr_name,$link_point,$chr_start,$chr_end,$score) = ();
    my @out = ();

    while(($study,$analysis,$chr_name,$link_point,$chr_start,$chr_end,$score) = $sth->fetchrow_array()) {
      last unless defined $chr_start;
      
      my $link_obj = Bio::EnsEMBL::Linkage->new();
      $link_obj->study($study);
      $link_obj->analysis($analysis);
      $link_obj->chr_name($chr_name);
      $link_obj->link_point($link_point);
      $link_obj->start($chr_start);
      $link_obj->end($chr_end);
      $link_obj->score($score);
      
      push @out, $link_obj;
    }
    return \@out;
}

1;
