# Supplementary material for: Statistical Viewer: a tool to upload and integrate linkage and association data as plots displayed within the Ensembl genome browser
Source: BMC Bioinformatics. 2005 Apr 12;6:95. doi: 10.1186/1471-2105-6-95 (PMC1087836; doi:10.1186/1471-2105-6-95)
Supplement: Additional File 5 — The source code for Bio::EnsEMBL::GlyphSet::FineLODplot BioPerl module [file 1471-2105-6-95-S5.rtf]

######################################################################
#                                                                    #
# Ensembl module for Bio::EnsEMBL::GlyphSet::finelodplot             #
#                                                                    #
# Maintained by Hong Xu <hxu@chg.duhs.duke.edu>				   # 
# Center for Human Genetics Bioinformatics Core          		   #
# Duke University Medical Center                                     #
#                                                                    #
#                                                                    #
# You may distribute this module under the same terms as perl itself #
#                                                                    #
# History:      2003-03-31  add x-axis & legend  -hxu                #
#               2003-04-08  add code to handle no linkage  -hxu      #
#               2003-04-16  change passing parameters  -hxu          #
#               2003-05-08  add analysis selection handle  -hxu      #
#               2003-05-23  add code to handle negative score -hxu   #
#               2004-01-03  adapt from lodplot module -hxu           #
#                                                                    #
######################################################################

package Bio::EnsEMBL::GlyphSet::finelodplot;
use strict;
use vars qw(@ISA);
use Bio::EnsEMBL::GlyphSet;
@ISA = qw(Bio::EnsEMBL::GlyphSet);
use POSIX;
use Sanger::Graphics::Glyph::Rect;
use Sanger::Graphics::Glyph::Poly;
use Sanger::Graphics::Glyph::Text;
use Sanger::Graphics::Glyph::Line;
use Sanger::Graphics::Glyph::Space;
use Sanger::Graphics::Glyph::Circle;
use Sanger::Graphics::Bump;

sub init_label {
    my ($self) = @_;
    return if( defined $self->{'config'}->{'_no_label'} );
    my $label = new Sanger::Graphics::Glyph::Text({
        'text'      => 'Stat. Score',
        'font'      => 'Small',
        'absolutey' => 1,
    });
    $self->label($label);
}

sub _init {
    my ($self) = @_;

    # only draw once - for the plus strand
    # return unless ($self->strand() == 1);

    # get the slice of chromosome 
    my $slice           = $self->{'container'};
    my $chr_nm          = $slice->chr_name();
    my $chrlen          = $slice->get_Chromosome()->length();
    my $vclen           = $slice->length();
    my $vcsta           = $slice->chr_start();
    my $vcend           = $slice->chr_end();
    
    return if ($vclen < 1000);    # don't want plot for very short sequences


    # get config options
    my $Config          = $self->{'config'};
    my $im_width        = $Config->image_width();
    my $study           = $Config->{'study'};
    my $lana            = $Config->{'analysis'};
    my $scl             = 200;
    my @clr             = ();
    push @clr, 'red';
    push @clr, 'black';
    push @clr, 'blue';
    push @clr, 'green';
    push @clr, 'cyan';
    push @clr, 'gray60';
    push @clr, 'maroon';
    push @clr, 'navy';
    push @clr, 'pink';
    push @clr, 'orange';
    
    return if ($study eq '');
    
    # fetch the linkage data for this slice
    my $lka = $slice->adaptor()->db()->get_LinkageAdaptor();
    my $links;
    if ( $lana ) {
      $links    = $lka->fetch_all_by_study_Slice_analysis($slice, $study, $lana);
    }
    else {
      $links = $lka->fetch_all_by_study_Slice($slice, $study);
    }

    # if no linkage result on the slice
    if (scalar(@{$links}) == 0) {
        $self->push(
            new Sanger::Graphics::Glyph::Text({
                'x'         => 100,
                'y'         => 20 ,
                'height'    => $Config->texthelper->height('Small'),
        'font'      => 'Small',
        'text'      => "There is no linkage point for study - $study on this region!",
                'colour'    => 'red',
                'absolutey' => 1,
                'absolutex' => 1,
            })
        );
    return;
    }
      
    
    # group linkage results
    my $max_score       = 0;
    my $min_score       = 0;
    my %analysis        = ();
    foreach my $link (@$links) {
        if ( $link->score() > $max_score ) {
            $max_score = $link->score();
        }
        if ( $link->score() < $min_score ) {
            $min_score = $link->score();
        }
        push( @{$analysis{$link->analysis()}}, $link );
    }
    my $ymax            = ceil( $max_score );
    my $ymin            = floor( $min_score );
    my $maxy            = $ymax - $ymin;

    
    #################################
    # Draw the linkage result
    #################################
    my $kc = 1;
    foreach my $key (sort keys %analysis) {
        my $pntn  = 1;
        my $plink = shift @{$analysis{$key}};
        my $type = $lka->get_type_by_study_analysis($plink->study(), $key);
        my $zm = $self->zmenu($plink,$vcsta);
        $self->push(
           new Sanger::Graphics::Glyph::Circle({
               'x'         => $plink->start(),
               'y'         => (1 - ($plink->score() - $ymin)/ $maxy) * $scl,
               'radius'    => 2,
           'absolutewidth' => 1,
           'filled'      => 1,
           'colour'    => $clr[$kc],
               'absolutey' => 1,
               'zmenu'  => $zm,
            })
        );
        if ( (defined $self->{'config'}->{'_label'}) && $lana && (scalar(@$lana) == 1) ) {
            $self->push(
                new Sanger::Graphics::Glyph::Text({
                    'x'         => $plink->start(),
                    'y'         => (1 - ($plink->score() - $ymin)/ $maxy) * $scl - 10,
                    'height'    => $Config->texthelper->height('Tiny'),
            'font'      => 'Tiny',
            'text'      => "$pntn",
                    'absolutey' => 1,
                    'zmenu'  => $zm,
                })
            );
            $pntn++;
        }
    
    foreach my $link ( @{$analysis{$key}} ) {
        my $zmn = $self->zmenu($link,$vcsta);
        $self->push(
        new Sanger::Graphics::Glyph::Circle({
            'x'         => $link->start(),
            'y'         => (1 - ($link->score() - $ymin)/ $maxy) * $scl,
                    'radius'    => 2,
                'absolutewidth' => 1,
                'filled'      => 1,
            'colour'    => $clr[$kc],
            'absolutey' => 1,
            'zmenu'  => $zmn,
                })
            );
            if ( (defined $self->{'config'}->{'_label'}) && $lana && (scalar(@$lana) == 1) ) {
                $self->push(
                    new Sanger::Graphics::Glyph::Text({
                        'x'         => $link->start(),
                        'y'         => (1 - ($link->score() - $ymin)/ $maxy) * $scl - 10,
                        'height'    => $Config->texthelper->height('Tiny'),
                'font'      => 'Tiny',
                'text'      => "$pntn",
                        'absolutey' => 1,
                        'zmenu'  => $zmn,
                    })
                );
                $pntn++;
            }
            if ($type ne 'dot' ) {
            $self->push(
            new Sanger::Graphics::Glyph::Line({
                'x'         => $plink->end(),
                'y'         => (1 - ($plink->score() - $ymin)/ $maxy) * $scl,
                'width'     => $link->start() - $plink->end(),
                'height'    => ($plink->score() - $link->score()) / $maxy * $scl,
                'colour'    => $clr[$kc],
                'absolutey' => 1,
                    })
                );
            }
        else {
            $self->push(
            new Sanger::Graphics::Glyph::Line({
                'x'         => $plink->end(),
                'y'         => (1 - ($plink->score() - $ymin)/ $maxy) * $scl,
                'width'     => $link->start() - $plink->end(),
                'height'    => ($plink->score() - $link->score()) / $maxy * $scl,
                'colour'    => $clr[$kc],
                'absolutey' => 1,
            'dotted'    => 1,
                    })
                );
            }
            $plink  = $link;
        }
    $kc++;
    }
    

    #################################
    # Draw the lod score coordinate
    #################################
    $self->push(
        new Sanger::Graphics::Glyph::Line({
            'x'         => 0,
            'y'         => 0,
            'width'     => 1,
            'height'    => $scl,
            'colour'    => $clr[1],
            'absolutey' => 1,
        })
    );

    for (my $i =  $ymin; $i <= $ymax; $i++) {
        $self->push(
            new Sanger::Graphics::Glyph::Line({
                'x'         => -3,
                'y'         => (1 - ($i - $ymin)/ $maxy) * $scl ,
                'width'     => 3,
                'height'    => 0,
                'absolutey' => 1,
                'absolutex' => 1,
            })
        );
        $self->push(
            new Sanger::Graphics::Glyph::Text({
                'x'         => -7,
                'y'         => (1 - ($i - $ymin)/ $maxy) * $scl ,
                'height'    => $Config->texthelper->height('Tiny'),
        'font'      => 'Tiny',
        'text'      => "$i",
                'absolutey' => 1,
                'absolutex' => 1,
            })
        );
    }


    #################################
    # Draw the one lod score drop down line
    #################################
    if ($max_score >= 1) {
      $self->push(
        new Sanger::Graphics::Glyph::Line({
            'x'         => 0,
            'y'         => (1 - ($max_score - 1 - $ymin)/$maxy) * $scl,
            'width'     => $vclen,
            'height'    => 0,
            'colour'    => $clr[0],
            'absolutey' => 1,
    'dotted'    => 1,
        })
      );
    }


    #################################
    # Draw legend
    #################################
    $kc = 1;
    my ($X, $Y) = (0,0);
    my $new_scl = $scl + 20;
    my $LEG_S   = 60;
    my $BOX_H   = 20;
    my $BOX_W   = 20;
    my $COL_N   = 2;

    $self->push(
        new Sanger::Graphics::Glyph::Text({
            'x'         => 0,
            'y'         => $new_scl,
        'height'    => $Config->texthelper->height('Small'),
        'font'      => 'Small',
          'text'      => 'Legend',
        'absolutey' => 1,
        'absolutex' => 1,
        })
    );
    foreach my $key (sort keys %analysis) {
        $self->push(
            new Sanger::Graphics::Glyph::Circle({
                        'x'         => $LEG_S + ($im_width - $LEG_S - 10) * $X / $COL_N + 10,
        'y'         => $new_scl + $Y * $BOX_H + 12,
                        'radius'    => 3,
                'absolutewidth' => 1,
                'filled'      => 1,
        'colour'    => $clr[$kc],
        'absolutey' => 1,
        'absolutex' => 1,
            })
         );
        $self->push(
            new Sanger::Graphics::Glyph::Text({
                        'x'         => $LEG_S + ($im_width - $LEG_S - 10) * $X / $COL_N + $BOX_W + 12,
        'y'         => $new_scl + $Y * $BOX_H + 8,
        'height'    => $Config->texthelper->height('Small'),
        'font'      => 'Small',
        'text'      => $key,
        'colour'    => $clr[$kc],
        'absolutey' => 1,
        'absolutex' => 1,
            })
         );
    $kc++;
    $X++;
    if ($X == $COL_N) {
        $X = 0;
        $Y++;
        }
    }
}

sub zmenu {
  my ($self, $f, $vcs ) = @_;
  my $zmenu = { 
     'caption'   => "Seq feature: " . $f->link_point,
     '01:Study: ' . $f->study => '',
     '02:Analysis: ' . $f->analysis => '',
     '03:Chr' . $f->chr_name . ': ' . ($f->start + $vcs) . '-' . ($f->end + $vcs) => '',
     '04:Score: ' . $f->score  => '',
  };
  return $zmenu;
}
           
1;
