# Supplementary material for: Statistical Viewer: a tool to upload and integrate linkage and association data as plots displayed within the Ensembl genome browser
Source: BMC Bioinformatics. 2005 Apr 12;6:95. doi: 10.1186/1471-2105-6-95 (PMC1087836; doi:10.1186/1471-2105-6-95)
Supplement: Additional File 9 — User manual for uploading Linkage or other statistical data to be plotted in a display panel called "Statistic View" within the Contig- and Cyto- view genome Browser pages of a local implementation of Ensembl. [file 1471-2105-6-95-S9.doc]

#
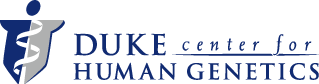


# Bioinformatics Core Resource

# User’s Guide to Uploading

# CHG Statistical Data into local Ensembl

The CHG Statistical data (linkage / association data) upload service page is the gateway for importing statistical analysis data into our local implementation of the Ensembl Genome Annotation System (www.ensembl.org). Once the analysis data is uploaded into Ensembl, we can view the analysis data as a 2-dimension plot within the context of the human genome annotation browser. The inclusion of such data is a valuable tool to integrate statistical analysis data with other public genome annotation information in order to facilitate the identification and prioritization of candidate complex human disease susceptibility genes.

This user’s guide contains detailed instructions for loading statistical data (such as linkage or association data as Lod scores or *p*-values) into our local Ensembl system. These instructions are grouped into three sections:

# Formatting the Data File for Upload

- 1. Required Fields …………………………………………………………… **2**
  2. Excel file format …………………………………………………………… **2**
  3. Tab-delimited text format ………………………………………………… **4**

# Login Procedure …………………………………………………………………….… 6

# The Upload Process …………………………………………………………..……… 8

**I. Formatting the Data for Upload**

# A. Required Fields

For the data to useful the file must have a minimum of 8 required fields. These are:

1. **Study**: It is used to define a data set, not the usual disease project. One study can have data across multiple chromosomes or in several analysis groups. You can include data from multiple studies into a single Excel spread sheet provided that all of the data is on the top worksheet. (*For an example please refer to the study display in the “1) study example” depicted in the screen shot in figure 3*)
2. **Analysis**: This is used to stratify data categories within a study. It can be different statistical analysis methods, or the different populations, etc. You can put several data categories into the same spreadsheet. (*An example of the analysis display is illustrated by the “2) Analysis example” in t in figure 3*).
3. **Link_point**: This could be marker name for linkage study. Or it could be SNP name for association study. *(Note: for multi-point analysis, some link points have no actual markers or SNPs associated with those points. Then you may make some fake names, such as “cM_10.02”*.)
4. **Score**: The statistical score for the Link_point.
5. **Chr_name**: The chromosome name, valid values are 1~22, X, Y for human.
6. **Chr_start**: The chromosome start location of the Link_point. Note it is in base pair unit, NOT kilo base pair.
7. **Chr_end**: The chromosome end location of the link_point. Note it is in base pair unit, NOT kilo base pair. (*Note: In the case of SNP markers, the Chr-end position is the same as the start location provided it is a true SNP and not multiple nucleotides or an indel*)
8. **Link_type**: This specifies the type of line used for connecting the Link_points in the plot: for example, “dot” is “dotted line ( )” for “two-point analysis” (see the screen shot below), and “line” is “solid line ( ) ” for “multi-point analysis”. If you don’t supply a value for this, the default will be “line”. If you place the value “point” in this field, it will draw scattered points without drawing a connection line.

**B. Excel File Format**

It is often convenient for analysts to use Microsoft Excel to store, manipulate and analyze data. For this reason the upload server is able to directly accept .xls files for upload via the Statistical Data Upload Service Web-based front end. Provided that the data file is similar in appearance to figure 1 the browse feature can be used to select the file for import and the upload process should be successful.

| **Study** | **Analysis** | **Link_Point** | **Score** | **Chr_name** | **Chr_start** | **Chr_end** | **Link_type** |
| --- | --- | --- | --- | --- | --- | --- | --- |
| ZTEST | MLOD-ALL | D5S2849 | 0.576 | 5 | 3559481 | 3559685 | line |
| ZTEST | MLOD-ALL | D5S1492 | 0 | 5 | 3845638 | 3845753 | line |
| ZTEST | MLOD-ALL | D5S2505 | 0.097 | 5 | 5983220 | 5983504 | line |
| ZTEST | MLOD-ALL | F13A1 | 0 | 5 | 6089317 | 6265901 | line |
| ZTEST | MLOD-ALL | D5S807 | 0.619 | 5 | 9377979 | 9378175 | line |
| ZTEST | MLOD-ALL | D5S2845 | 0.874 | 5 | 22827194 | 22827347 | line |
| ZTEST | MLOD-ALL | D5S2848 | 1.3108 | 5 | 27209712 | 27209927 | line |
| ZTEST | MLOD-ALL | D5S1470 | 0.0076 | 5 | 32938485 | 32938664 | line |
| ZTEST | MLOD-ALL | D5S1457 | 0.0216 | 5 | 42126019 | 42126115 | line |
| ZTEST | MLOD-ALL | D5S2500 | 0 | 5 | 60249704 | 60249857 | line |
| ZTEST | MLOD-ALL | D5S1501 | 0 | 5 | 78708155 | 78708263 | line |
| ZTEST | MLOD-ALL | D5S1725 | 0 | 5 | 89457554 | 89457738 | line |
| ZTEST | MLOD-ALL | D5S1462 | 0.101 | 5 | 96844702 | 96844903 | line |
| ZTEST | MLOD-ALL | D5S1453 | 0.026 | 5 | 108183988 | 108184151 | line |
| ZTEST | MLOD-ALL | D5S1505 | 0.289 | 5 | 119499926 | 119500182 | line |
| ZTEST | MLOD-Cauc | D5S2849 | 0.401 | 5 | 3559481 | 3559685 | line |
| ZTEST | MLOD-Cauc | D5S1492 | 0.787 | 5 | 3845638 | 3845753 | line |
| ZTEST | MLOD-Cauc | D5S2505 | 0.129 | 5 | 5983220 | 5983504 | line |
| ZTEST | MLOD-Cauc | F13A1 | 0.593 | 5 | 6089317 | 6265901 | line |
| ZTEST | MLOD-Afro-A | D5S2849 | 0.501 | 5 | 3559481 | 3559685 | line |
| ZTEST | MLOD-Afro-A | D5S1492 | 0.887 | 5 | 3845638 | 3845753 | line |
| ZTEST | MLOD-Afro-A | D5S2505 | 0.0947 | 5 | 5983220 | 5983504 | line |
| ZTEST | MLOD-Asian | D5S2849 | 0.751 | 5 | 3559481 | 3559685 | line |
| ZTEST | MLOD-Asian | D5S1492 | 0.087 | 5 | 3845638 | 3845753 | line |
| ZTEST | MLOD-Asian | D5S2505 | 0.147 | 5 | 5983220 | 5983504 | line |
| ZTEST | MLOD-Asian | F13A1 | 0.593 | 5 | 6089317 | 6265901 | dot |
| ZTEST | HetLod-ALL | D5S2849 | 0.876 | 5 | 3559481 | 3559685 | dot |
| ZTEST | HetLod-ALL | D5S1492 | 0.397 | 5 | 3845638 | 3845753 | dot |
| ZTEST | HetLod-ALL | D5S2505 | 0.077 | 5 | 5983220 | 5983504 | point |
| ATEST | NonPara-ALL | rs62849 | 0.876 | 5 | 39259481 | 3559685 | point |
| ATEST | NonPara-ALL | rs87542 | 0 | 5 | 392559488 | 3845753 | point |

Figure 1. An example excel file of data from a study called ZTEST containing the minimum necessary fields in a format suitable for upload into the CHG’s local Ensembl using the Statistical Data Upload Server

Notes:

1. The data sheet should be the first worksheet in the Excel file.
2. There should not have any merged fields in the spreadsheet.
   1. New data sets are simply concatenated in rows below the first set with the value for the field changed. (See lower rows in Figure 1)

**C. Tab-Delimited Text File Format**

The Statistical data upload server is able handle the upload of tab-delimited text files as some user may generate their data in UNIX or simply prefer or have a need to use text files.

Trouble shooting: Sometimes special characters are inadvertently created by “cutting-and-pasting”. Should this occur the server may be unable to successful uploading Excel spreadsheets. This problem can be easily be overcome by saving the spreadsheet as “Text (Tab delimited)” file.

The fields in figure 2 are identical to those shown the Excel file. See the example below:


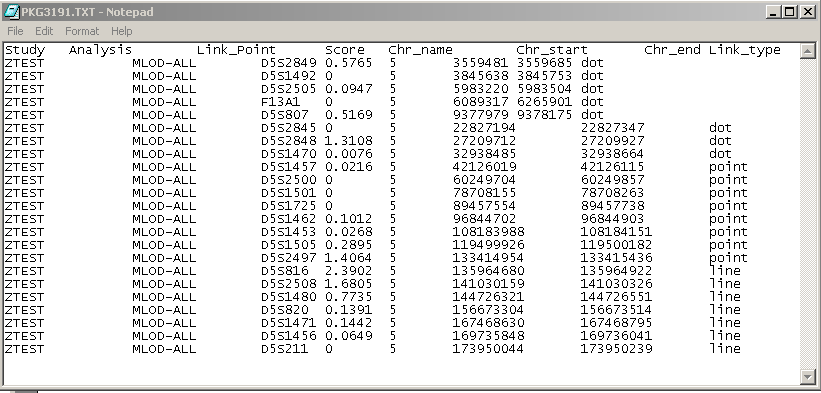


Figure 2. The same data as in figure 1, but saved as a tab-delimited text (.txt) file

Figure 3. Screen shot of the Statistical data display panel integrated into the “ Contig View” page of our local Ensembl genome browse

**2. Login**

To use the CHG Ensembl-DAS upload service, you must first log into the CHG Data page. This page is password protected: the user ID is “chg”, the password is “bi0inf”. Please follow the steps below:

- Go to local Ensembl-DAS site: http://genominator2.duhs.duke.edu


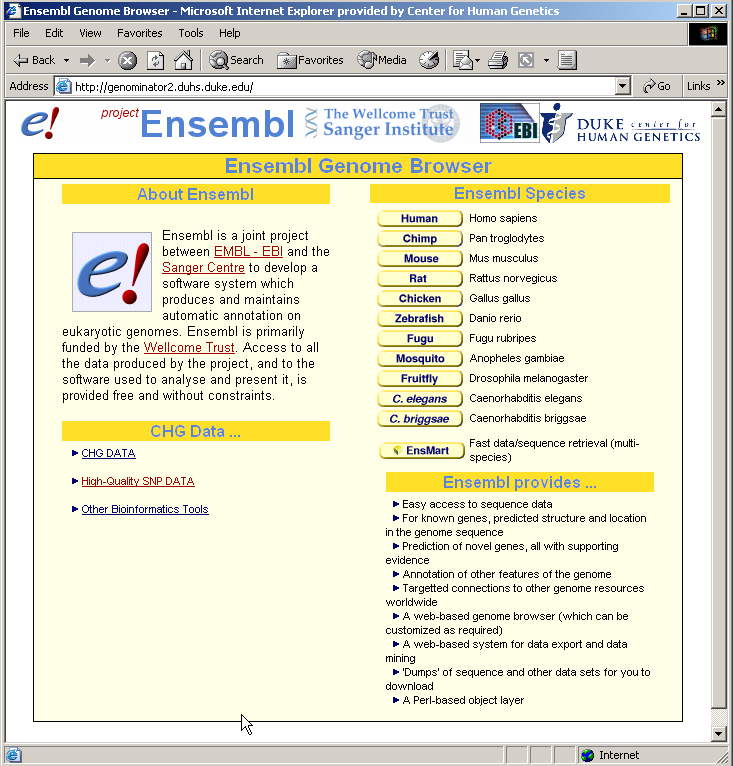


- Click the link – “CHG DATA” under “CHG Data …” section on the lower left of the CHG Ensembl home page. This will pop up a “Security alert” page, please click “Yes” button to proceed.


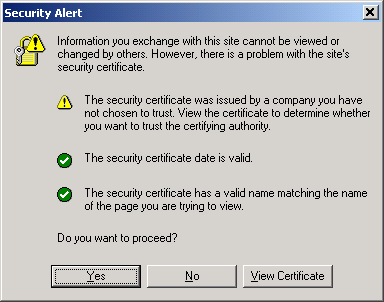


- Once you have clicked on the “Yes” button, a new window will appear requesting a user ID and password.
- Please type in user ID and password, then click “OK” button.
- Checking the save password box will allow you to avoid retyping the password when you login in the future


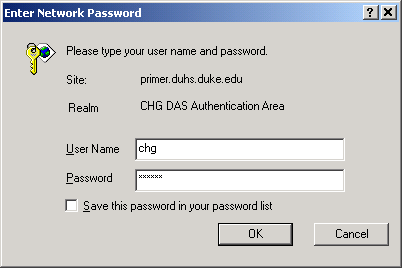


- At this point you are viewing the CHG internal DAS page. Please put the cursor over the drop-down menu – “Data”, then click the first menu entry – “Upload Linkage Data”.

**3. The Upload Process**

Once you have compiled the data so that it conforms to the proper format criteria and contains the eight minimum required fields (column), you can upload linkage / association data through the web-interface. To use the “statistical data upload service” to upload the data file, you simply need to provide the appropriate information for the three input fields. The email address of the user is necessary so that the server can return the upload results to the user. Next the browse button tool must be used to specify the data file to be uploaded and the path specifying its location. As mentioned in section 1, this file can be either an Excel file or a text file in which fields are delimited by tab (\t). Finally, please use the File Type pull-down menu to select format option that corresponding to the data file you are uploading.


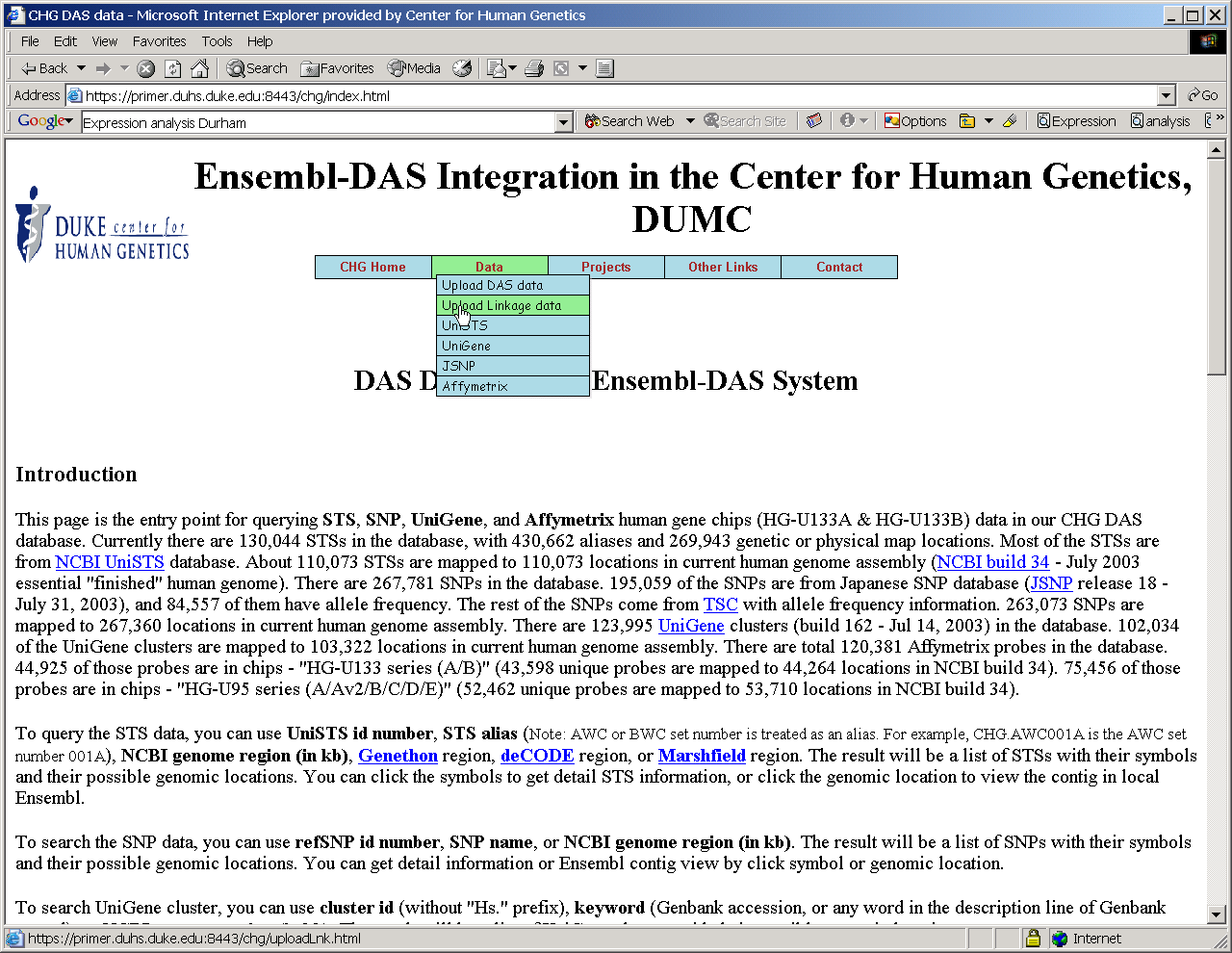


- Then this will take you to the “Statistical data upload” page.


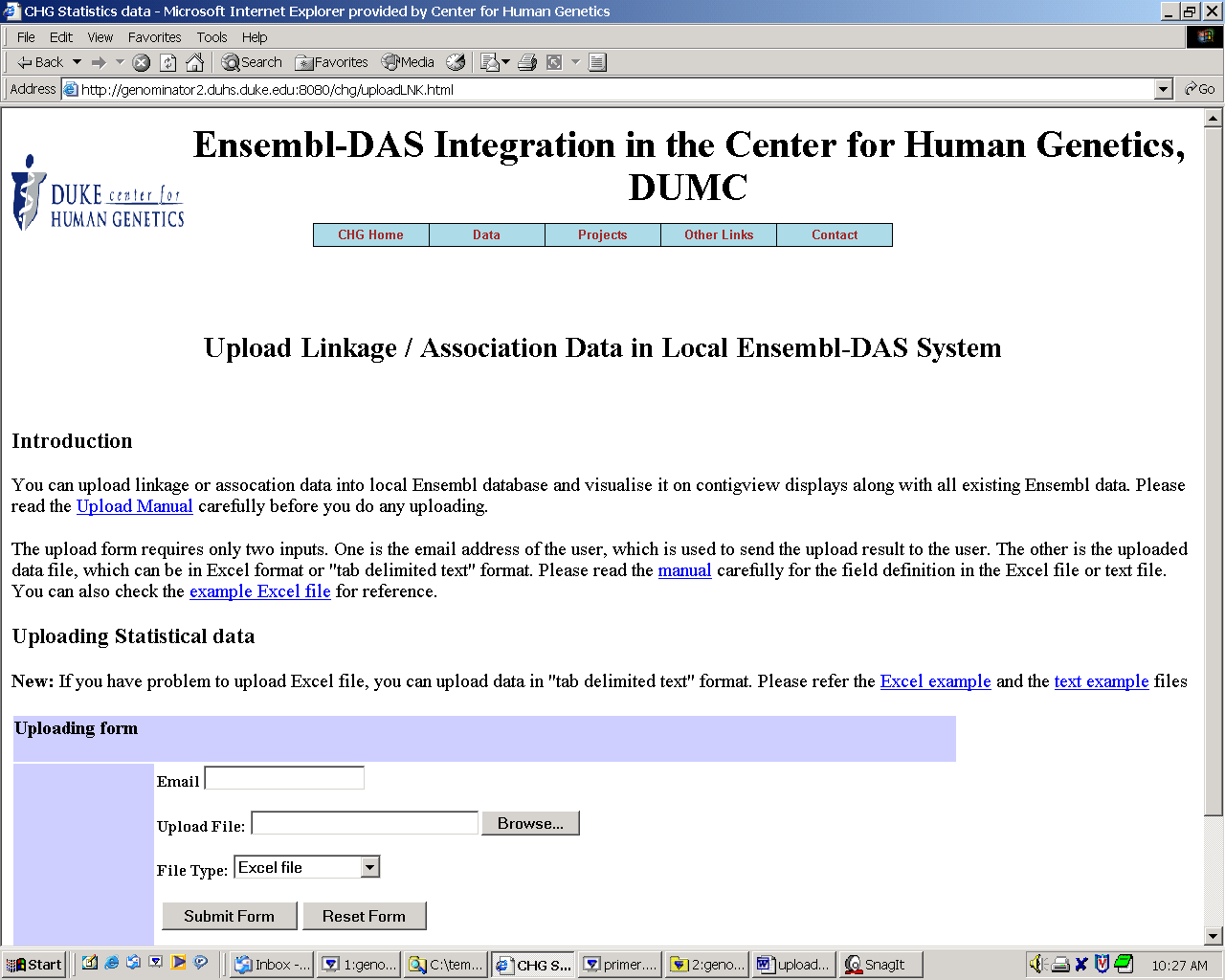


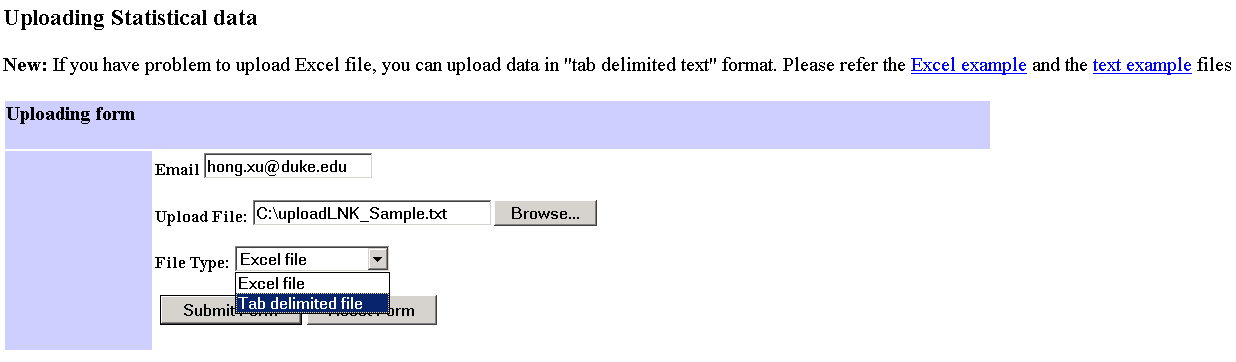


Email address

upload file path & name

choose file format

After you click “Submit Format” button, the program will return a message that the result file is sending to your email address.


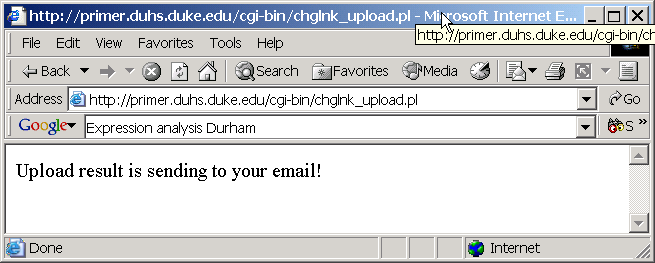


If you check your email, you should see message like this:


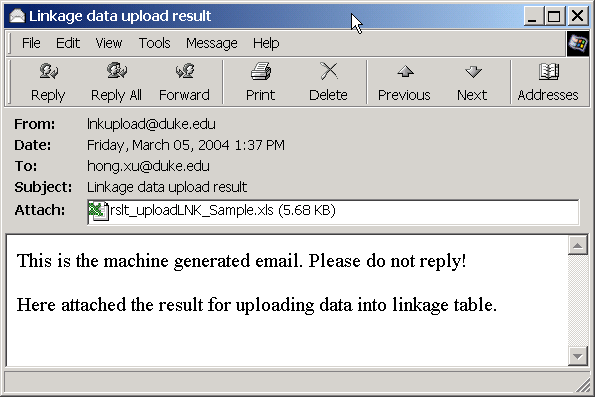


Then the result file will tell summarize the date and indicate whether the data load was successful or not. Should the upload fail the reason will be given.

| **Study** | **Analysis** | **Link_point** | **Score** | **Upload_info** |
| --- | --- | --- | --- | --- |
| ZTEST | MLOD-ALL | D5S2849 | 0.5765 | Upload successful |
| ZTEST | MLOD-ALL | D5S1492 | 0 | Upload successful |
| ZTEST | MLOD-ALL | D5S2505 | 0.0947 | Upload successful |
| ZTEST | MLOD-ALL | F13A1 | 0 | Upload successful |
| ZTEST | MLOD-ALL | D5S807 | 0.5169 | Upload successful |
| ZTEST | MLOD-ALL | D5S2845 | 0 | Upload successful |
| ZTEST | MLOD-ALL | D5S2848 | 1.3108 | Upload successful |
| ZTEST | MLOD-ALL | D5S1470 | 0.0076 | Upload successful |
| ZTEST | MLOD-ALL | D5S1457 | 0.0216 | Upload successful |
| ZTEST | MLOD-ALL | D5S2500 | 0 | Upload successful |
| ZTEST | MLOD-ALL | D5S1501 | 0 | Upload successful |
| ZTEST | MLOD-ALL | D5S1725 | 0 | Upload successful |
| ZTEST | MLOD-ALL | D5S1462 | 0.1012 | Upload successful |
| ZTEST | MLOD-ALL | D5S1453 | 0.0268 | Upload successful |
| ZTEST | MLOD-ALL | D5S1505 | 0.2895 | Upload successful |
| ZTEST | MLOD-ALL | D5S2497 | 1.4064 | Upload successful |
| ZTEST | MLOD-ALL | D5S816 | 2.3902 | Upload successful |
| ZTEST | MLOD-ALL | D5S2508 | 1.6805 | Upload successful |
| ZTEST | MLOD-ALL | D5S1480 | 0.7735 | Upload successful |
| ZTEST | MLOD-ALL | D5S820 | 0.1391 | Upload successful |
| ZTEST | MLOD-ALL | D5S1471 | 0.1442 | Upload successful |
| ZTEST | MLOD-ALL | D5S1456 | 0.0649 | Upload successful |
| ZTEST | MLOD-ALL | D5S211 | 0 | Upload successful |

If you go to the local Ensembl web page on chromosome 5 and choose study “ZTEST”, it should look like this:

**
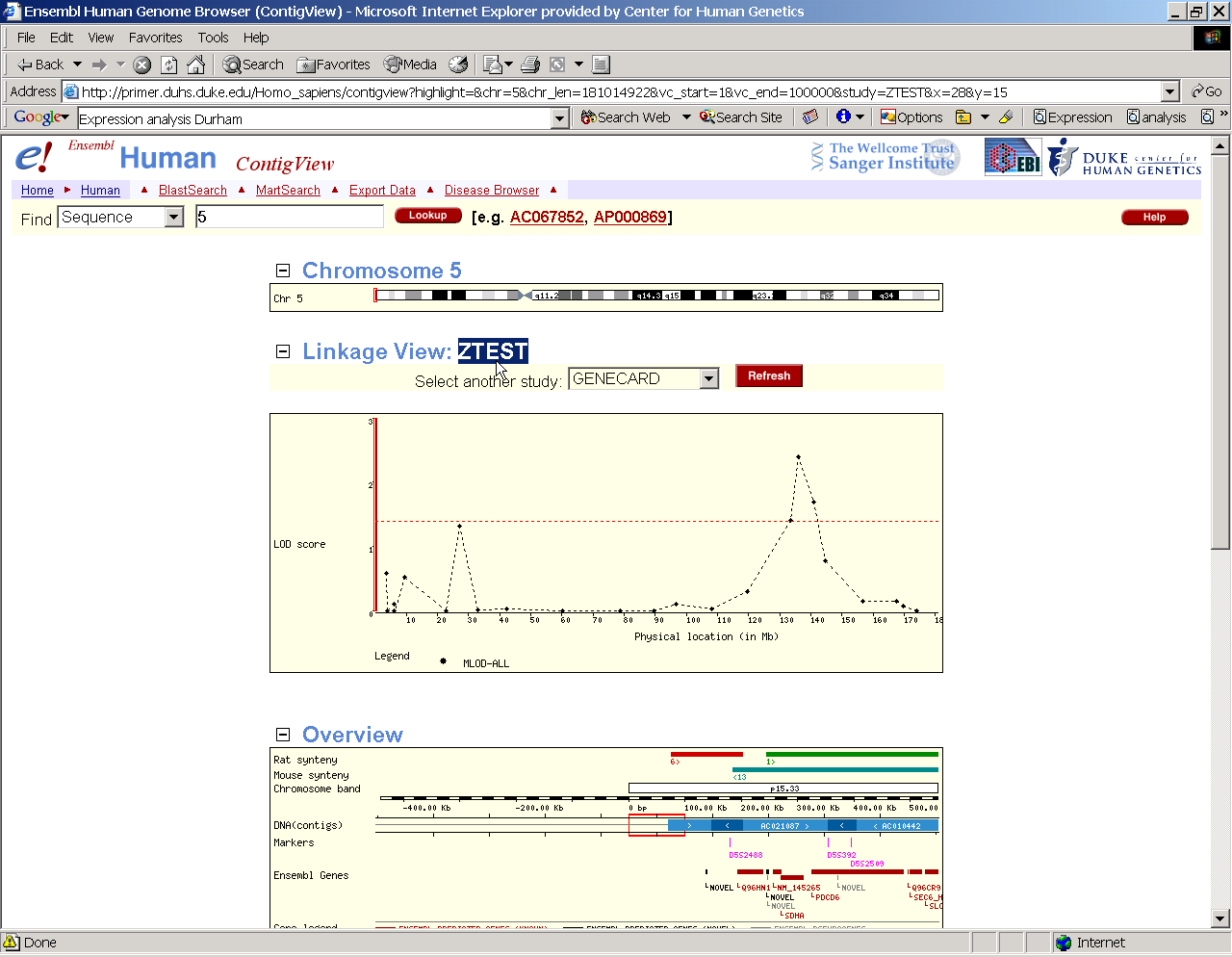
**
