# Supplementary material for: Statistical Viewer: a tool to upload and integrate linkage and association data as plots displayed within the Ensembl genome browser
Source: BMC Bioinformatics. 2005 Apr 12;6:95. doi: 10.1186/1471-2105-6-95 (PMC1087836; doi:10.1186/1471-2105-6-95)

#
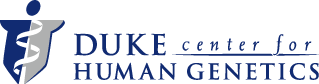


# Bioinformatics Core Resource

# The CHG

# Ensembl-DAS Upload Server

# Users’ Guide

# Introduction

The CHG Ensembl-DAS upload service web page provides a user-friendly gateway for importing experimental or other data into the DAS server. This server offers several advantages over the one provided at www.ensembl.org, as it allows researchers to upload and append data they generated, in the form or either MS Excel or tab-delimited text files, into the local DAS-Ensembl genome annotation system. Once experimental data is imported into the DAS system, this data along with public genome annotation information many other types of data from other sources can be visualized as DAS feature tracks within the context of the public human genome sequence assembly and annotation. By providing a basis for a bioinformatics infrastructure, this integration and data storage system help researches synthesize large amounts of data efficiently. Thus a local implementation of the Ensembl databases, genome browser and annotation system, and supplemented the distributed annotation system, is an invaluable tool for geneticists employing multiple approaches to help identify and prioritize candidate complex disease susceptibility genes for follow up analysis.

This user’s guide contains detailed instructions for loading experimental data into the DAS system. These instructions are grouped into six sections:

**Contents**

**Getting Started** ……………………………………………………………………………………. 2

**The Login Procedure** …………………………………………………..………………………… 3

**Excel File Format** …………………………………………………………………………….…… 6

**The Upload Procedure**……………………………………………………………………….…… 9

**Updating data** …………………………………………………………………………………..… 11

**Appendix**

**Locating the genomic position of a feature** …………………………………………………… 13

**l. Getting Started**

1. First bring up the [CHG local Ensembl web](http://genominator2.duhs.duke.edu/) server page on your web brower
   1. This web page can be accessed the in either of two ways:
      1. Directly by entering the URL: <http://genominator2.duhs.duke.edu/>
      2. Indirectly through the CHG internal home page: https://wwwchg.duhs.duke.edu


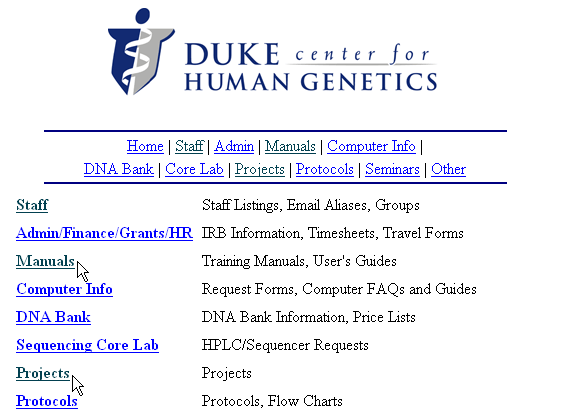


Note: *Clicking on the Manual page will bring up a directory of many other important Manuals and User Guides. The Manual page has a link to the Bioinformatics Manual Page:*

<https://wwwchg.duhs.duke.edu/manuals/bioinformatics/bioinformatics.html>

Figure 1. The CHG internal home page. Clicking on “Projects” takes you to the  [Internal Project Page](https://wwwchg.duhs.duke.edu/chg_projects.html) : ([https://wwwchg.duhs.duke.edu/ chg_projects.html](https://wwwchg.duhs.duke.edu/chg_projects.html))

- Click on the Project Hyperlink to bring up the  [Internal Project Page](https://wwwchg.duhs.duke.edu/chg_projects.html) shown below

­­­­­­­­­­­­­­
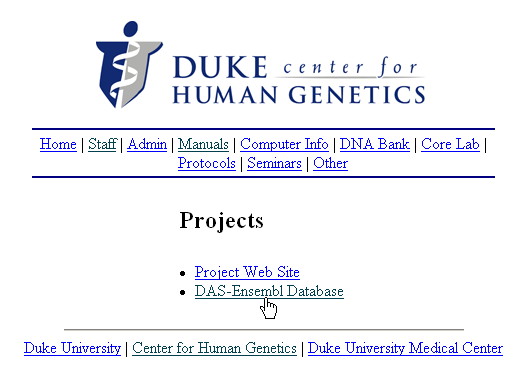


Figure 2: The CHG Project Page provides a link to DAS Ensembl web server

- **The CHG data page must be accessed from the Local DAS-Ensembl home page**


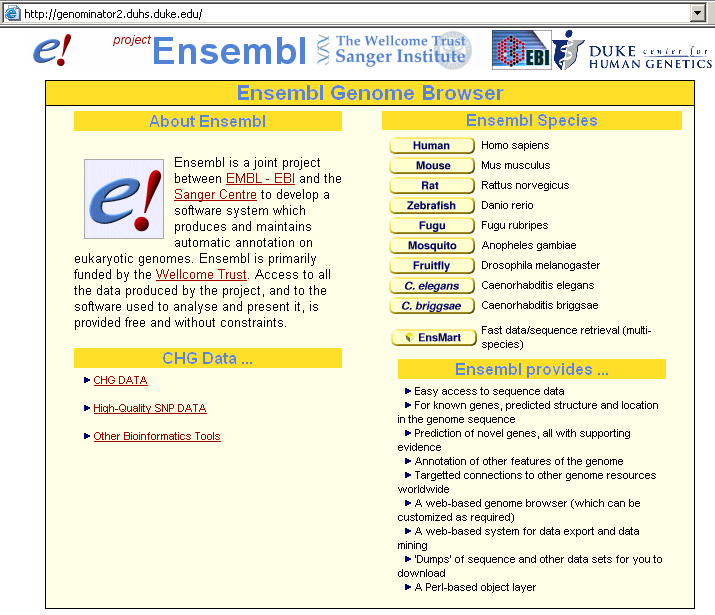


Click Here to get to

The DAS integration page

**Figure 3: Homepage for local implementation of open-source DAS-enabled-Ensembl genome annotation system at the CHG.** The local implementation of DAS-Ensembl (shown running on Domain 2 of the the Duke CHG Sunfire 12K “Genominator2” high end server is the foundation of the bioinformatics infrastucture at the Duke CHG. DAS-Ensembl allows researchers to view private bioinformatics data, statistical data, and laboratory generated experimental data and public annotation in the context of the latest NCBI human genome sequence assembly (currently Build 34), The CHG DAS Ensembl Home Page provides a link to CHG private data page

To use the CHG Ensembl-DAS upload service, you have to go through the local DAS-Ensembl home page (fig. 3) and then through the CHG Data page

- **First click on the “CHG Data” link shown in the lower portion of the CHG-Ensembl home page.**

**ll. The Login Procedure**

To use the CHG Ensembl-DAS upload service, you have to go through the CHG Data page. This page is password protected so that a security alert window (see fig. 3) will appear when you try to access the server. **Click on the “CHG Data” link shown in the lower right-hand corner of the page to bring up the Security Pop-up window shown below:**


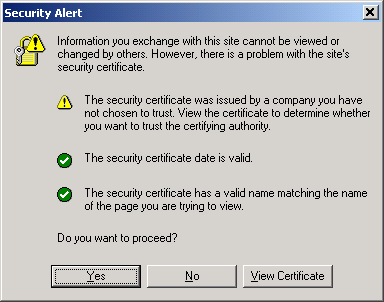


Figure 4. The security alert window

- To proceed, click the “Yes” button and a new page will pop-up that requests the user ID and password.


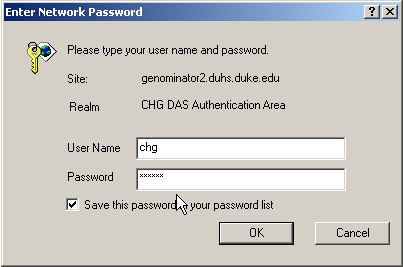


Figure 5. security access window for CHG internal data.

- Type in the user ID and the password and click “OK” to access the DCHG Internal DAS Integration Page (shown in fig. 6).


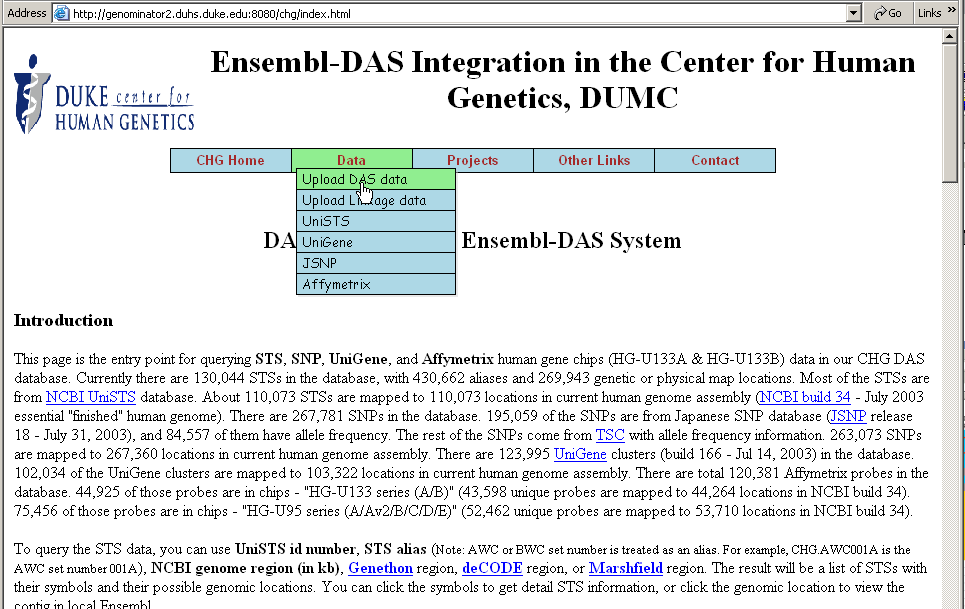


### Figure 6: The DCHG internal DAS integration page. Besides allowing web-based queries of local data on features such as SNPs, STSs, and Unigene, this page provides access to our enhanced upload server developed to simplify the process of up-loading locally-derived data into the DAS server (into a *mySQL* database that may be queried directly to extract data meeting specified criteria if needed).

### To upload correctly formatted data (See table 1) that is contained within the top sheet of an MS-Excel file click on the “Upload DAS Data” option in the pull down menu, as shown in figure 6, to bring up the “DAS upload Service” page shown below in figure 7.


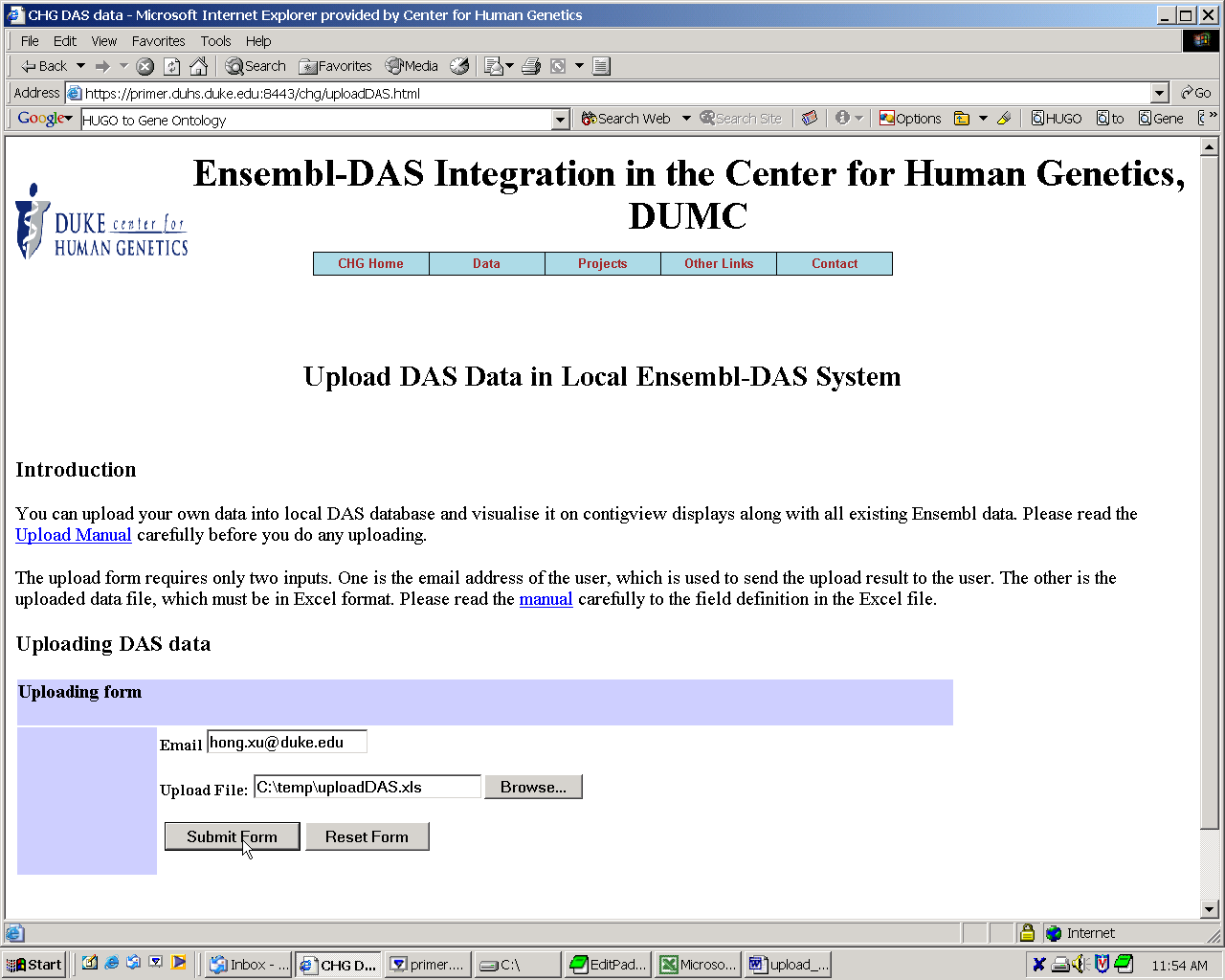


**Figure 7. The Web-based upload service page** allows a file to be uploaded from the users disk drive using the browse tool.

### Uploaded data is stored on the DAS server so that it can be displayed with a project-specific feature track in the local Ensembl genome browser once the server (genominator2) is rebooted. Once you have uploaded the data initially it need not be reloaded as we have software that automates the process of mapping existing features to subsequent releases of the assembly.

**lll. Excel File Format**

To upload data into DAS-Ensembl, it must have 7 required fields. These are:

1. **ID**: It could be standard gene symbol, SNP id (either public rs number or Celera hcv number), microarray probe id, marker name, etc.
2. **User**: Please use your CHG computer login user name for consistency.
3. **Project**: Since the DAS track for each project has to be pre-configured, the project name has to be precisely the same as the name in DAS configuration. Please see the following table for the project name.
   1. Note that you have data for multiple projects in the same spreadsheet (but NOT in the same cell, as merged rows are not tolerated).
   2. If you cannot find a pre-defined project name (see table 2) for your project, please send an email to [hong.xu@duke.edu](mailto:hong.xu@duke.edu) to initialize the project prior to uploading your data.
4. **Chr**: The chromosome name should be 1~22, and X, Y for human.
5. **Strand**: The chromosome strand value should be either 1 or –1. Other values will be set to 1 in the upload process.
6. **Start**: The chromosome start location of the feature. Note it is in base pair unit, NOT kilo base pair.
7. **End**: The chromosome end location of the feature. Note it is in base pair unit, NOT kilo base pair. Note: For SNP, it will be the same as the start location if it’s not multiple nucleotides insert/delete SNP.

**Table 1. A sample of a correctly formatted Excel file. To upload data into DAS-Ensembl, it must have 7 required fields (shown in a light yellow background) and 0 or more optional fields (shown in a light blue background).**

| **ID** | **User** | **Project** | **chr** | **strand** | **start** | **end** | **AccesionNumber** | **GeneSymbol** | **LocusLinkID** |
| --- | --- | --- | --- | --- | --- | --- | --- | --- | --- |
| 36753_at | adobra | CEGS | 19 | 1 | 59871298 | 59871447 | AF072099 | LILRB4 | 11006 |
| 925_at | adobra | CEGS | 19 | 1 | 18147412 | 18149898 | J03909 | IFI30 | 10437 |
| 39728_at | adobra | CEGS | 19 | 1 | 18149077 | 18149910 | J03909 | IFI30 | 10437 |
| 1062_g_at | adobra | CEGS | 11 | 1 | 117409513 | 117409811 | U00672 | IL10RA | 3587 |
| 40365_at | adobra | CEGS | 19 | 1 | 3114444 | 3114734 | M63904 | GNA15 | 2769 |
| 39982_r_at | adobra | CEGS | 8 | -1 | 16007338 | 16007812 | D13265 | MSR1 | 4481 |
| 36878_f_at | adobra | CEGS | 6 | -1 | 32673752 | 32675298 | M60028 | HLA-DQB1 | 3119 |
| 37200_at | adobra | CEGS | 1 | -1 | 158780434 | 158780955 | J04162 | FCGR3A | 2214 |
| 2092_s_at | adobra | CEGS | 4 | 1 | 89362255 | 89362661 | J04765 | SPP1 | 6696 |

Note: The data must be the first worksheet in the Excel file. Also, the spreadsheet must not contain any merged fields (columns)

*Note: as many optional fields as you like can be added as additional columns.*

Table 3. Diferrent projects on our local server. These can be appended simultaneously as long as the project name is supplied on a new row (essentially a record).

| **Project Name** | **Project Description** |
| --- | --- |
| **AAO** | Age at on-set project |
| **ALZ** | Alzheimer's disease project |
| **AMD** | Age-related macular degeneration project |
| **AUT** | Autism disease project |
| **AGENDA** | AGENDA project |
| **PD** | Parkinson's disease project |

After the optional data are loaded into database, the field name will become a property name, and the field value will become a property value.

As an example of how uploaded data is accessed from the browser, please see the data supplied in the first row (record) of the Excel sample file shown in table 1, will appear in a feature report page as shown in figure 8. This data can be accessed within the Ensembl genome browser Contig View page.

*Note :If you embed a “URL address” into a the text containing the value within an optional field, the program will build a hyper-link connecting the value to the “URL address”. For example:*

- To bring up the record page for a feature, you simply “mouse-over” the feature in the DAS track to display a pop-up window (that provides most essential annotation)and then slide the cursor to click the hyper-link (labeled “more information”) at the bottom of the pop-up window as shown in figure 8.

**Figure 8. A screen shot of a small section of the Detailed View panel in Ensembl’s Contig View Page** showing features that were uploaded in a project-specific DAS track displayed within the context of the Genome Assembly. Mousing over the feature caused the pop-up box with the project heading to appear (A). Moving the selection cursor (the hand) down to the bottom of the box followed by clicking will bring up the Data Report page for the feature, to show all fields for the record that were uploaded into the DAS server.


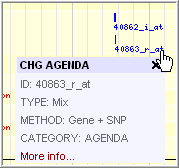

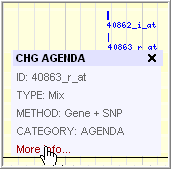


**B**

**A**


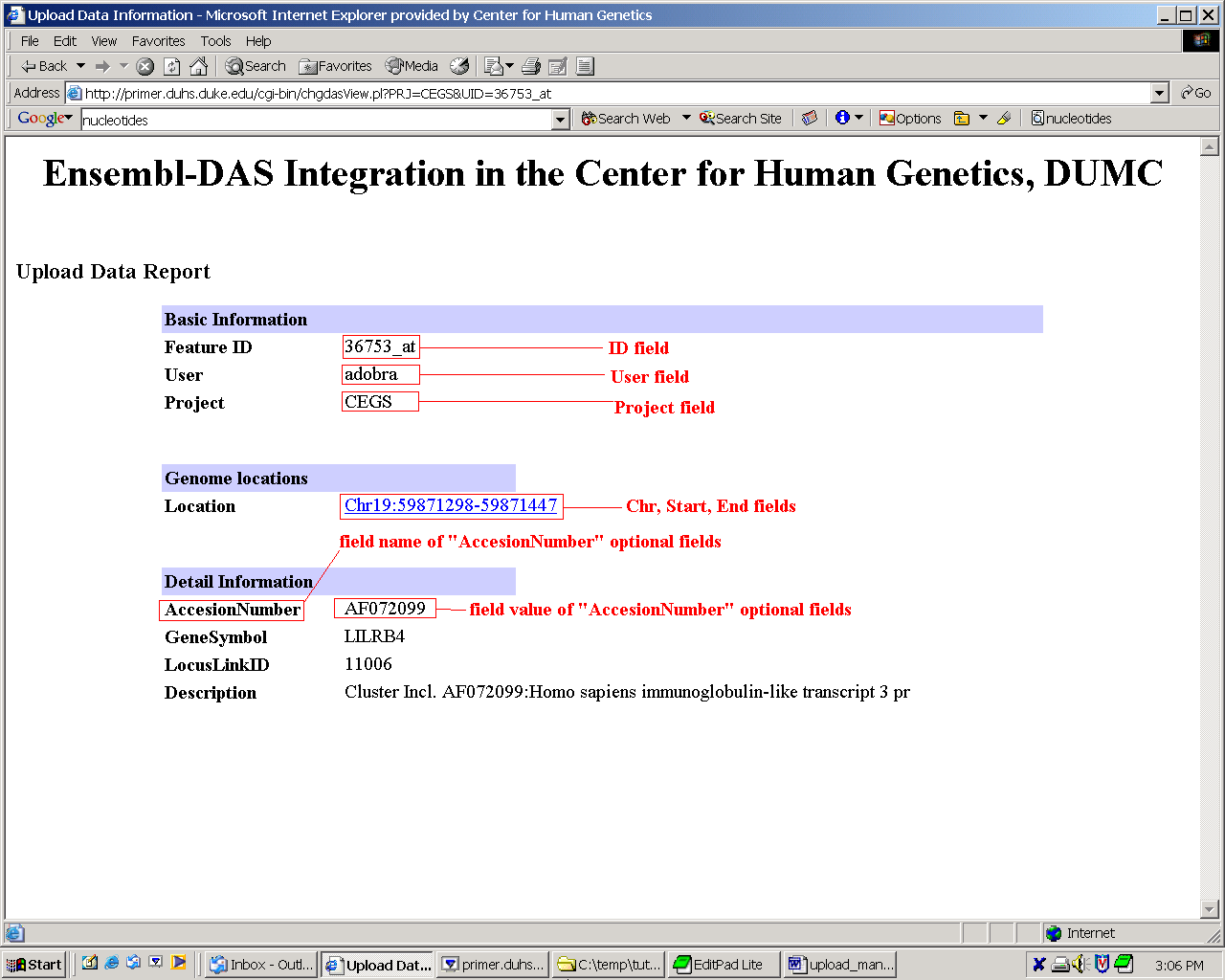


**Figure 9. An example of a feature report html page** containing annotation (values) for a record pertaining to feature 36753-at, an Affymetrix probe set ID for a differentially expressed gene in a microarray study done for the CEGS project . This data was uploaded into the CHG DAS Server for integration into the local Ensembl genome browser. This and other reports are accessed through the Detailed View in Ensembl’s Contig- and Cyto- view pages as illustrated in figure 7.

A unique feature report page contains all the information (values) supplied for the properties (fields heading columns) for each record (row) that was in the upload file


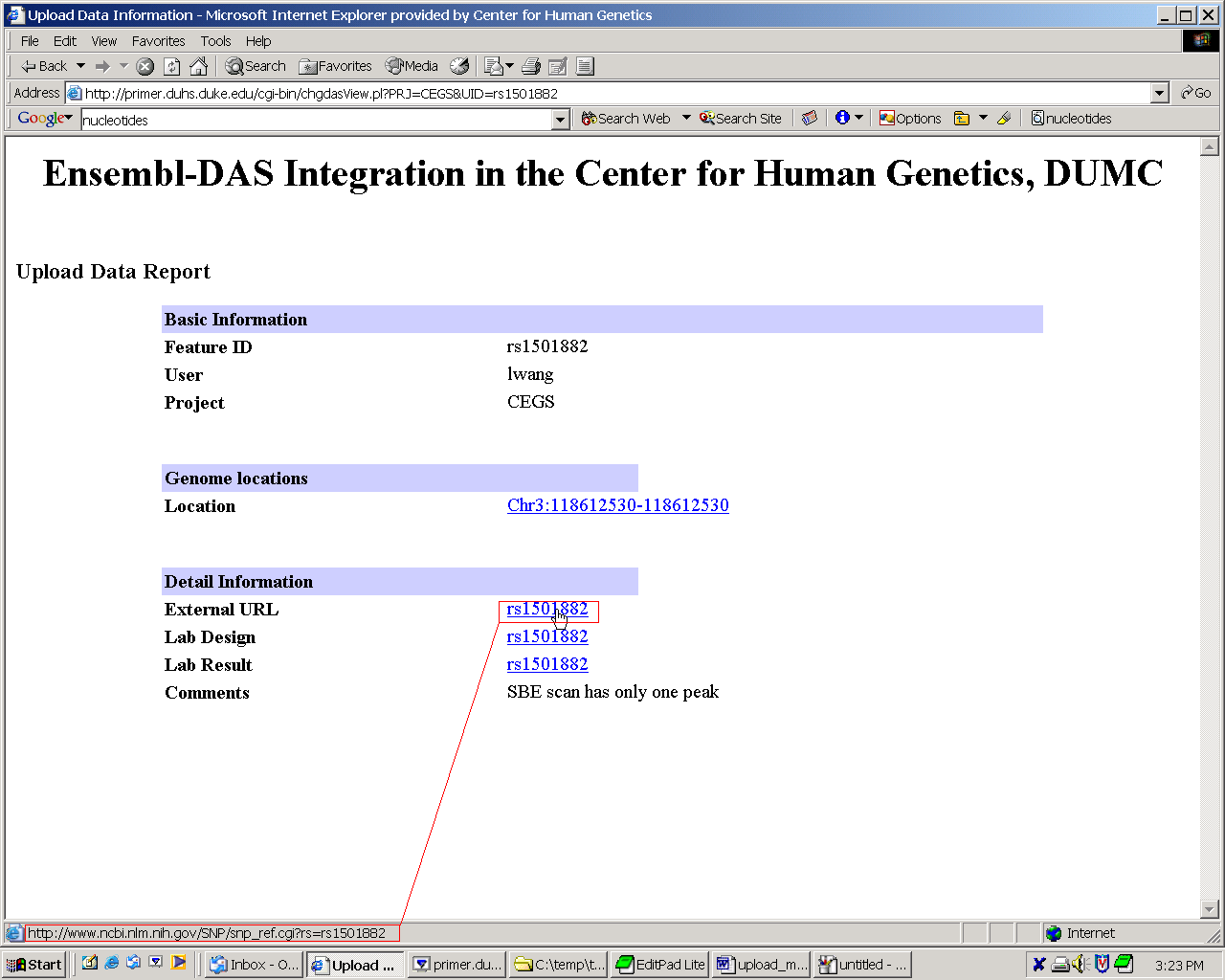


**Fig. 10. A data report page with hyper-links embedding in the values.** Upload files containing values for a field may include a hyper-link that can connecting to a URL location. Then, additional annotation on the feature can be brought up by clicking on the link. Shown is a SNP that contains a link to an external URL (in this case the dbSNP record) for which the address is displayed at the bottom of the browser window. A red line is draw in to emphasize the connection of the value to the URL address of this feature

**The Upload Procedure**

Once you have obtained the genomic location (with respect to the current build) for your DAS feature, you can upload the DAS feature through “DAS upload service page” for display in the Ensembl genome browser. If the feature is a marker for which you only have a genetic position, you can use the Get Map tool to easily get either the exact location or a close estimation. Get Maps retrieves the position from an internal database if the sequence could be mapped to the current genome assembly by ePCR or BLAT, otherwise Get Map determines the two closest flanking markers with known coordinates and calculates the approximate position by using linear interpolation.

- To upload the DAS features in Excel, you just need to put two inputs. One is the email address of the user, which is used to send the upload result to the user. The other is the uploaded data file, which must be in Excel format.


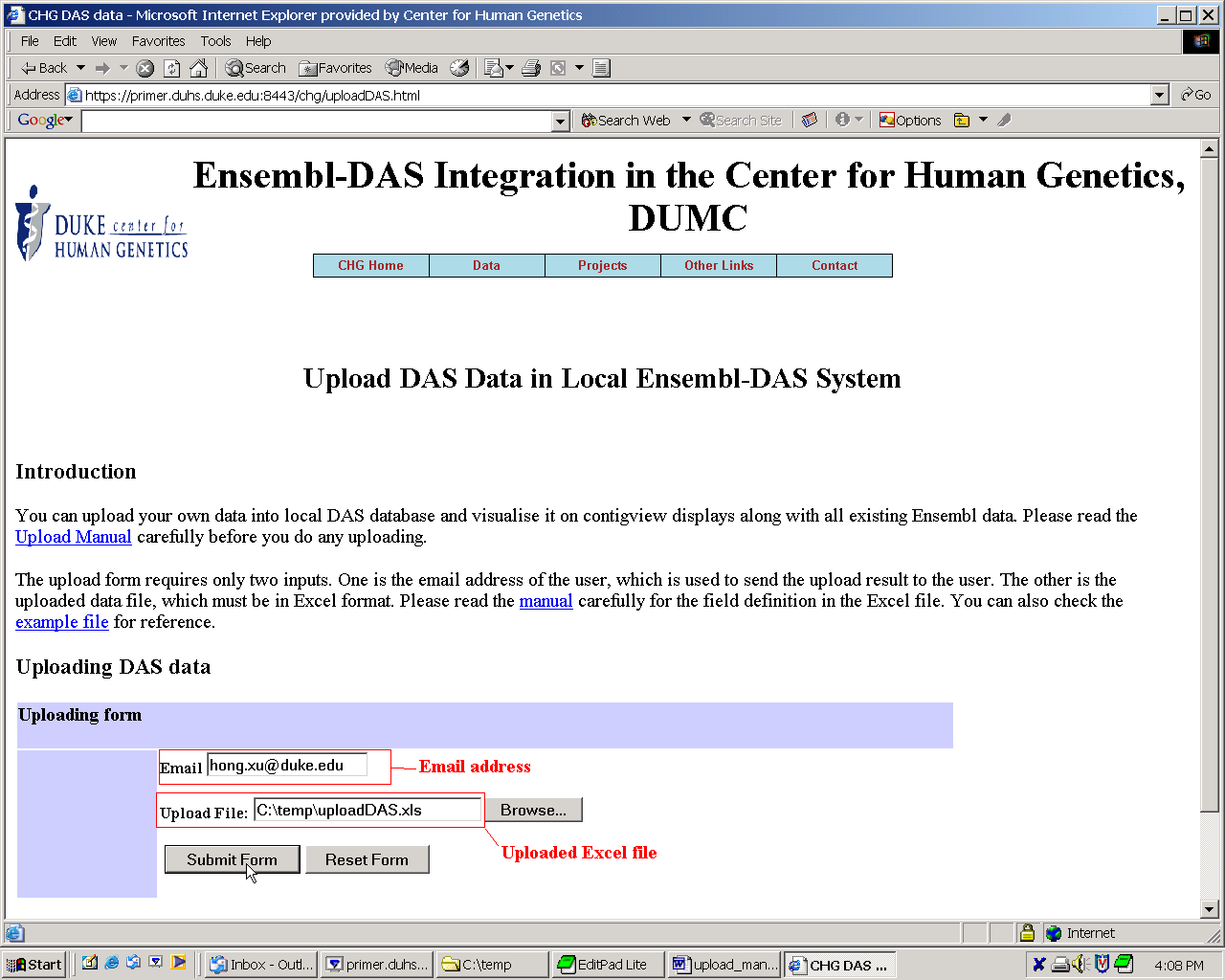


**Figure 11. The Duke CHG Internal DAS Upload Service Page.** Necessary information that must be supplied as input into the forms includes the email address of the user as well the path specifying the file and location that is selected by the browse tool

- Once you have supplied the email and file information click the “Submit Form” button
- The program will return a message indicating that a file containing the results of the upload is being sent to your email address.

If you check your email, you should see message like this:


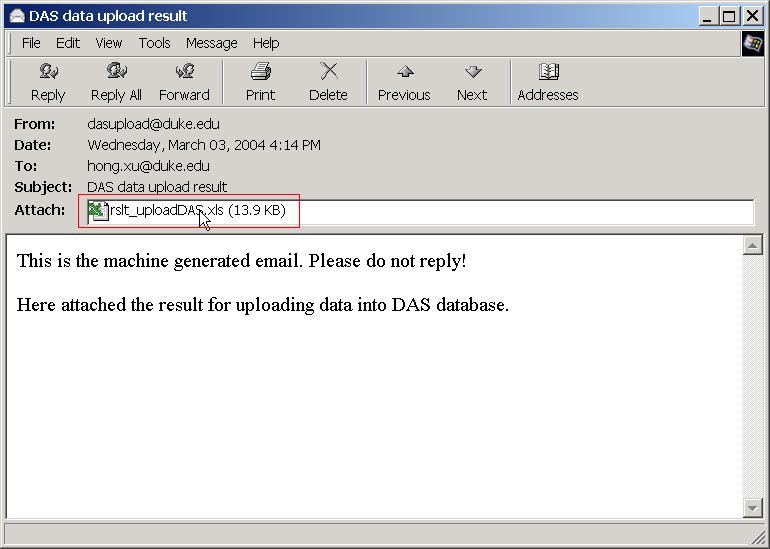


**Figure 12. The email message returned by the DAS Upload Server.** The message includes the results of the upload as an attachment

- The attached results file is an Excel will tell you whether the data load was successful or not. If the upload was unsuccessful, the reason for the failure will be indicated in the “Upload_info” column


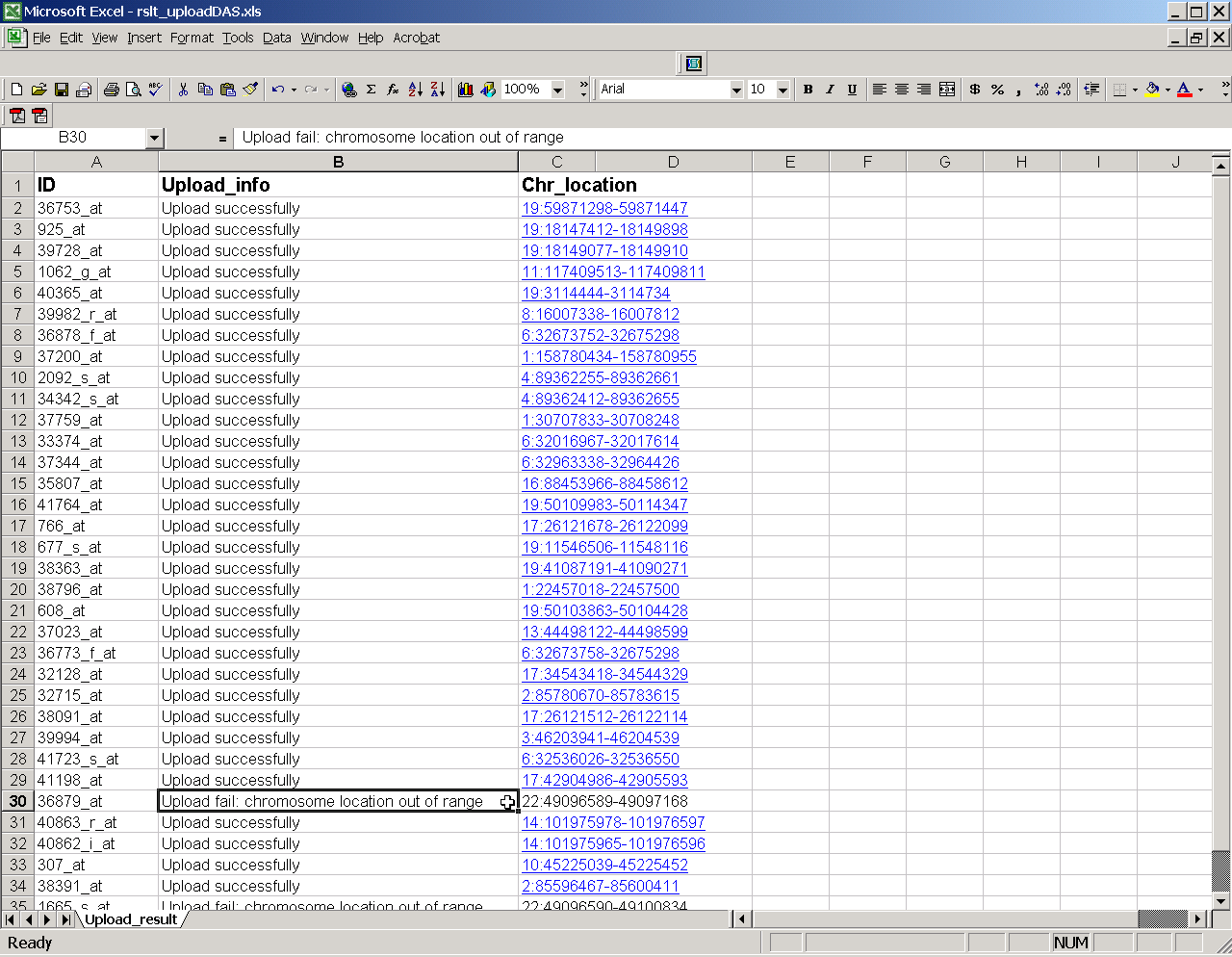


**Figure 13. An example of the results file returned by the DAS upload server following data submission.** Note that the record for the probe set ID feature in row 30 could not be mapped because the coordinates specify a position out of the range of chromosome 22.

**Update Process**

If you want to edit or append the information (such as “comments”, “Lab result”, etc.) of your uploaded feature, you can update the annotation for the feature using the same uploading service page. Since the uploaded feature is uniquely identified by the “ID”, “User”, and “Project” fields, you simply use the same “ID”, “User”, “Project”, and genome location fields (including “Chr”, “Strand”, “Start”, and “End” fields) to edit or update data in the optional fields. You can also append records for additional fields as well as supplement the annotation with new optional fields for the same “ID”, “User”, and “Project”. For this reason it is best to save the original updated file so it can be easily edited and uploaded to provide update information

For Example, for the first row data, we have updated its “LocusLink ID” value with NCBI LocusLink URL for ID #11006. We also have added another field as “GO: Molecular function”. The input like this:


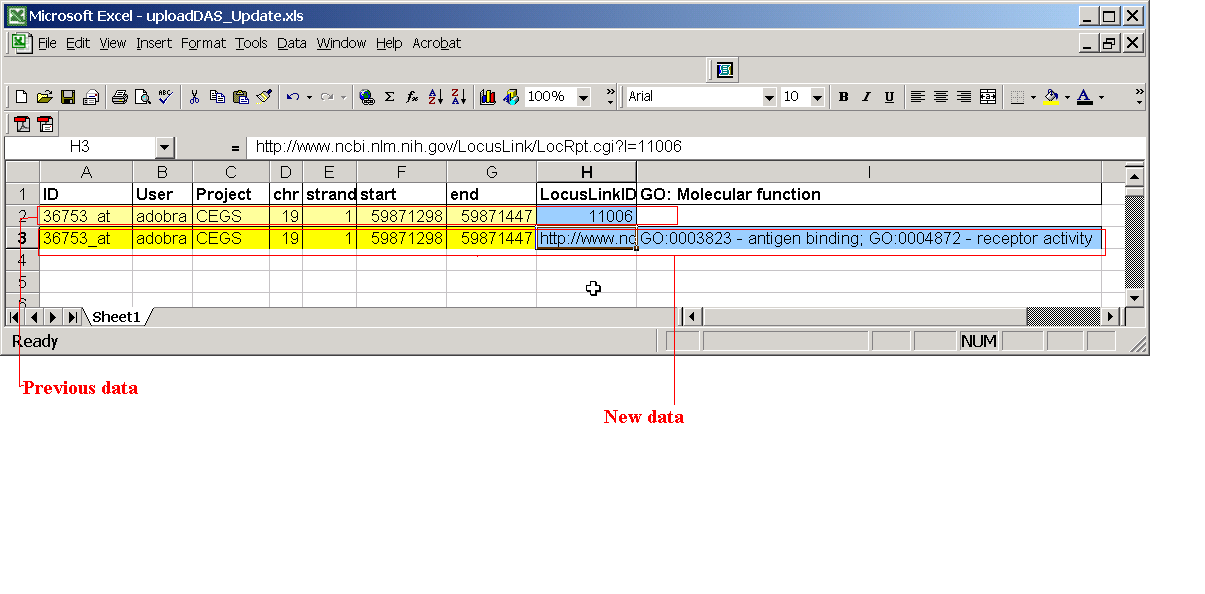


**Figure 14. An excel file with additional data to be edited or appended**


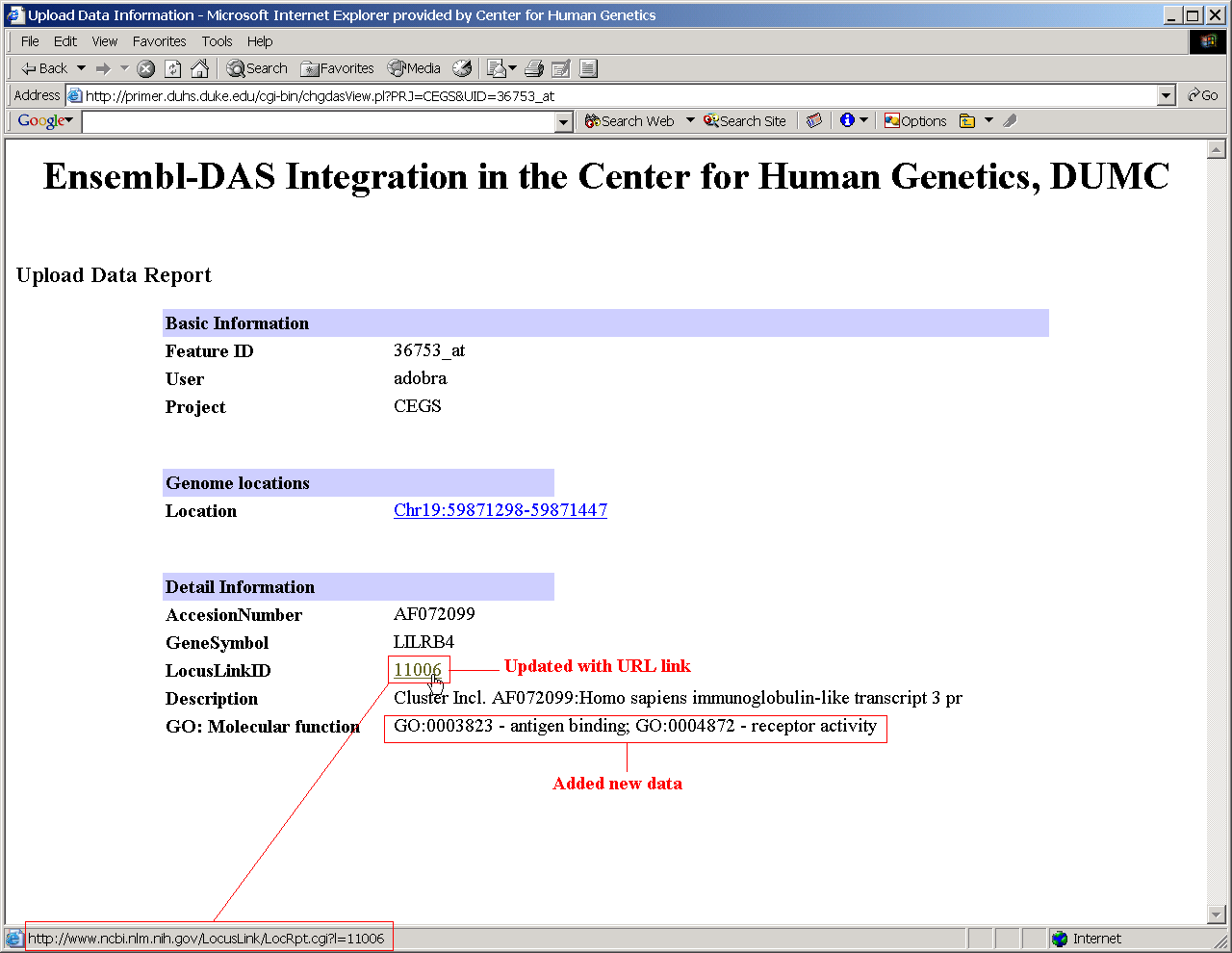


The DAS view page will now look like this:

**Figure 15. An updated version of the report page shown in figure 9** following the upload of the file shown above in fig.14.

**Appendix**

**Finding the Genomic Location for a Feature**

Before you upload your DAS feature, you have to find its genomic location (Note: please take note of the human genome assembly build version currently available on local Ensembl site. You must supply the bp coordinates that pertain to the assembly build that is in use by the browser). You can now use the Get Map tool if you have the genetic position of markers without sequence information. If you have the sequence information and the feature is not a mapped marker we suggest using either the UCSC BLAT server or the Ensembl BLAST server to locate your DAS feature.

- If you have a sequence (> 22bp), you can try UCSC BLAT server. It will quickly find sequences of 95% and greater similarity. But it’s less sensitive than BLAST.

<http://genome.ucsc.edu/cgi-bin/hgBlat?command=start&org=human>

- To use the UCSC BLAT server, type the DNA sequence (in FASTA format) into search text box and click “Submit” button:


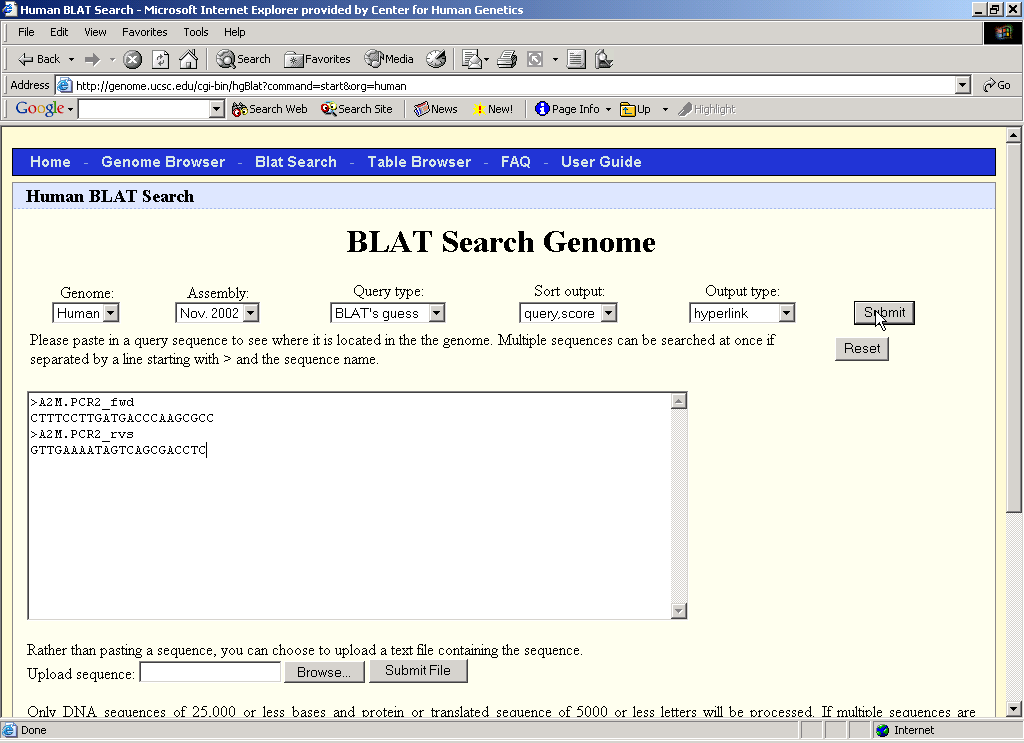


- This will generate a list of BLAT search results. You should choose the alignment with the best match. In some case, the best alignment is still a partial match because of the draft status of that genome region, you have to take extra caution. You may try other seuqence alignment server, such as Ensembl or NCBI BLAST server to find the right location.


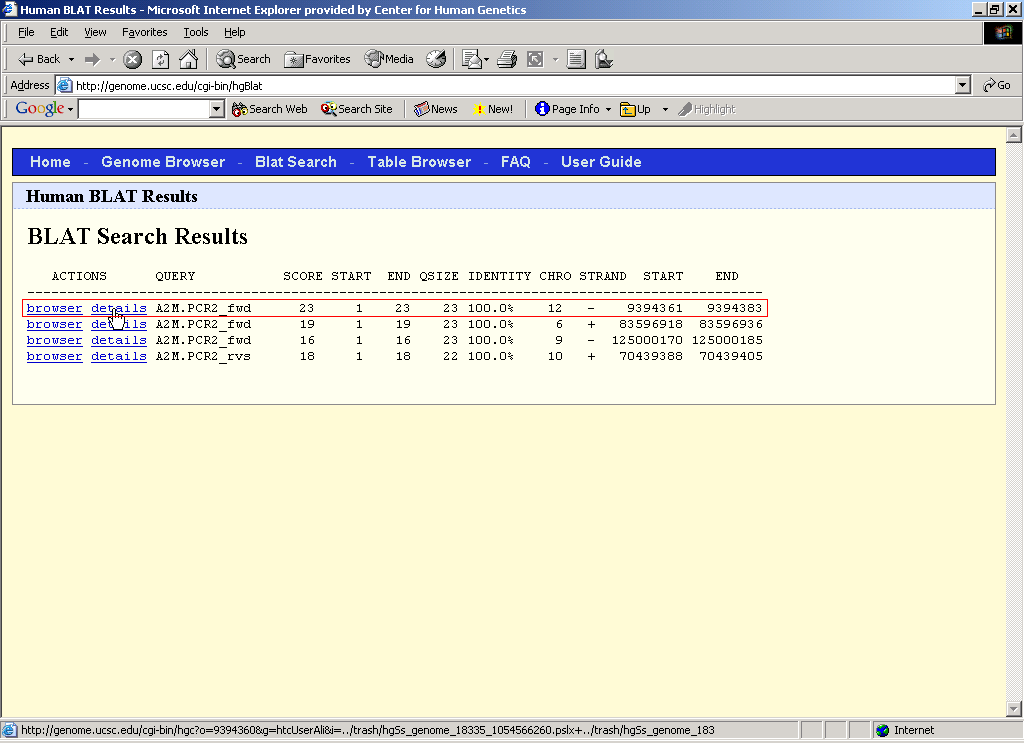


- If you couldn’t find the sequence location on UCSC BLAT server or the location is not correct, try Ensembl BLAST server. It’s more sensitive than BLAT. But it’s slower than BLAT search.

<http://www.ensembl.org/Homo_sapiens/blastview>

- You can upload sequence from file or paste sequence as FASTA format:


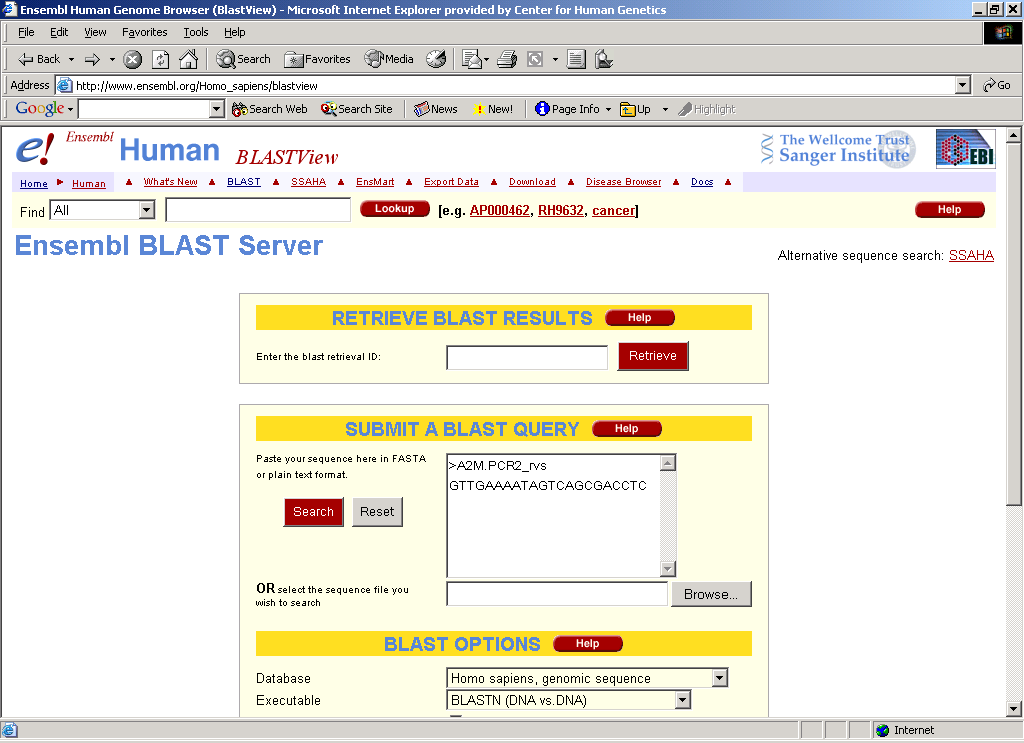


- Then you will get a page with BLAST retrieval ID.


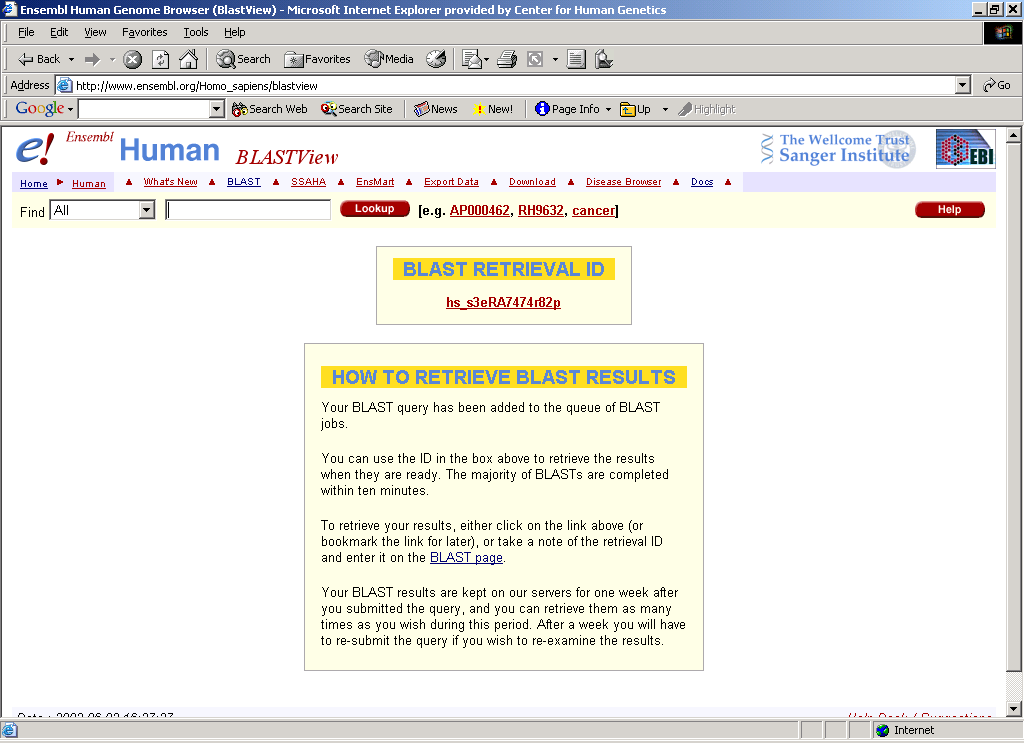


- After click the retrieval ID, you can the BLAST hits on chromosomes. The best hit is in a red box.


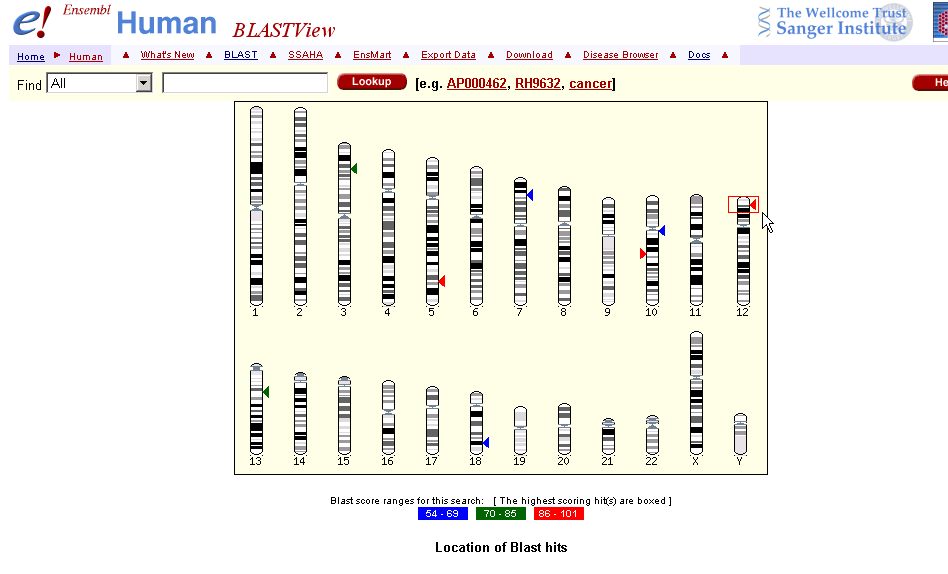


- By clicking the best BLAST hit, you can find a pop-up window with BLAST hit information.


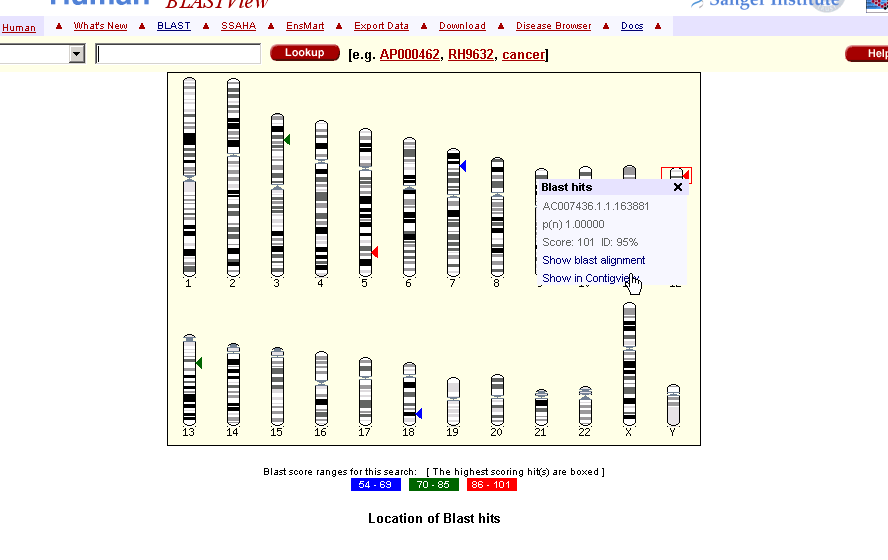


- By clicking “Show in Contigview”, you can see the BLAST hit in the Contigview page. If you move the mouse cursor over the BLAST hit, a pop-up window will show the genome location of the BLAST hit:


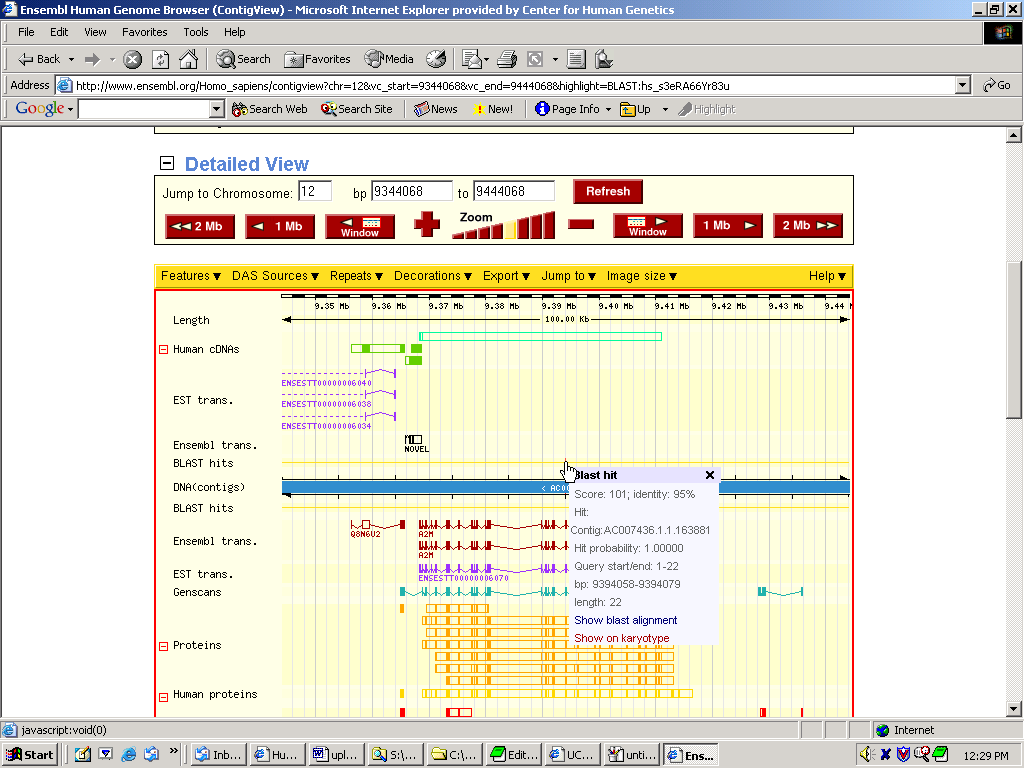

Supplement: Additional File 10 — The manual for using the Duke CHG enhanced upload server web interface to import data into MySQL database so that the features can be displayed as DAS-tracks showing annotation for experimental data within the context of the genome sequence assembly on a local implementation of Ensembl. [file 1471-2105-6-95-S10.doc]
