# Supplementary material for: Statistical Viewer: a tool to upload and integrate linkage and association data as plots displayed within the Ensembl genome browser
Source: BMC Bioinformatics. 2005 Apr 12;6:95. doi: 10.1186/1471-2105-6-95 (PMC1087836; doi:10.1186/1471-2105-6-95)
Supplement: Additional File 2 — The source code for Bio::EnsEMBL::Linkage [file 1471-2105-6-95-S2.rtf]

######################################################################
#                                                                    #
# Ensembl module for Bio::EnsEMBL::GlyphSet::lodplot             	   #
#                                                                    #
# Maintained by Hong Xu <hxu@chg.duhs.duke.edu>				   # 
# Center for Human Genetics Bioinformatics Core          		   #
# Duke University Medical Center                                     #
#                                                                    #
#                                                                    #
# You may distribute this module under the same terms as perl itself #
#                                                                    #
# History: 2003-03-31  add x-axis & legend  - hxu                    #
#                                                                    #
######################################################################


package Bio::EnsEMBL::GlyphSet::lodplot;
use strict;
use vars qw(@ISA);
use Bio::EnsEMBL::GlyphSet;
@ISA = qw(Bio::EnsEMBL::GlyphSet);
use Sanger::Graphics::Glyph::Rect;
use Sanger::Graphics::Glyph::Poly;
use Sanger::Graphics::Glyph::Text;
use Sanger::Graphics::Glyph::Composite;
use Sanger::Graphics::Glyph::Line;
use Sanger::Graphics::Glyph::Space;
use Sanger::Graphics::Bump;

sub init_label {
    my ($self) = @_;
    return if( defined $self->{'config'}->{'_no_label'} );
    my $label = new Sanger::Graphics::Glyph::Text({
        'text'      => 'LOD score',
        'font'      => 'Small',
        'absolutey' => 1,
    });
    $self->label($label);
}

sub _init {
    my ($self) = @_;

    # only draw once - for the plus strand
    return unless ($self->strand() == 1);

    # get the slice of chromosome 
    my $slice           = $self->{'container'};
    my $chr_nm = $slice->chr_name();
    my $chrlen          = $slice->get_Chromosome()->length();
    my $vclen           = $slice->length();

    # get config options
    my $Config          = $self->{'config'};
    my $im_width        = $Config->image_width();
    my $scl             = 200;
    my $highlights      = $self->highlights();
    my $cmap            = $Config->colourmap();
    my @clr             = ();
    push @clr, $cmap->id_by_name('red');
    push @clr, $cmap->id_by_name('black');
    push @clr, $cmap->id_by_name('blue');
    push @clr, $cmap->id_by_name('green');
    push @clr, $cmap->id_by_name('cyan');
    push @clr, $cmap->id_by_name('gray60');
    push @clr, $cmap->id_by_name('maroon');
    push @clr, $cmap->id_by_name('navy');
    push @clr, $cmap->id_by_name('pink');
    push @clr, $cmap->id_by_name('maroon');

    
    # fetch the linkage data for this chromosome
    my $lka = $slice->adaptor()->db()->get_LinkageAdaptor();
    my $links = $lka->fetch_all_by_chr_name($chr_nm);

    # if no linkage result on the chromosome
    if (scalar(@{$links}) == 0) {
        $self->push(
            new Sanger::Graphics::Glyph::Text({
                'x'         => 100,
                'y'         => 20 ,
                'height'    => $Config->texthelper->height('Small'),
'font'      => 'Small',
'text'      => "There is no linkage point on this chromosome!",
                'colour'    => $cmap->id_by_name('red'),
                'absolutey' => 1,
                'absolutex' => 1,
            })
        );
return;
    }
      
    
    # group linkage results
    my $max_score       = 0;
    my %analysis        = ();
    foreach my $link (@$links) {
        if ( $link->score() > $max_score ) {
            $max_score = $link->score();
        }
        push( @{$analysis{$link->analysis()}}, $link );
    }
    my $maxy            = int($max_score) + 1;

    
    #################################
    # Draw the linkage result
    #################################
    my $kc = 1;
    foreach my $key (sort keys %analysis) {
my $plink = shift @{$analysis{$key}};
        $self->push(
           new Sanger::Graphics::Glyph::Line({
               'x'         => $plink->start(),
               'y'         => (1 - $plink->score() / $maxy) * $scl,
               'width'     => $plink->start() - $plink->end(),
               'height'    => 0,
               'colour'    => $clr[$kc],
               'absolutey' => 1,
            })
        );
foreach my $link ( @{$analysis{$key}} ) {
    $self->push(
new Sanger::Graphics::Glyph::Line({
    'x'         => $link->start(),
    'y'         => (1 - $link->score() / $maxy) * $scl,
    'width'     => $link->start() - $link->end(),
    'height'    => 0,
    'colour'    => $clr[$kc],
    'absolutey' => 1,
                })
            );
    $self->push(
new Sanger::Graphics::Glyph::Line({
    'x'         => $plink->end(),
    'y'         => (1 - $plink->score() / $maxy) * $scl,
    'width'     => $link->start() - $plink->end(),
    'height'    => ($plink->score() - $link->score()) / $maxy * $scl,
    'colour'    => $clr[$kc],
    'absolutey' => 1,
                })
            );
            $plink  = $link;
        }
$kc++;
    }
    

    #################################
    # Draw the lod score coordinate
    #################################
    $self->push(
        new Sanger::Graphics::Glyph::Line({
            'x'         => 0,
            'y'         => 0,
            'width'     => 1,
            'height'    => $scl,
            'colour'    => $clr[1],
            'absolutey' => 1,
        })
    );

    for (my $i = 0; $i <= $maxy; $i++) {
        $self->push(
            new Sanger::Graphics::Glyph::Line({
                'x'         => -3,
                'y'         => (1 - $i / $maxy) * $scl ,
                'width'     => 3,
                'height'    => 0,
                'absolutey' => 1,
                'absolutex' => 1,
            })
        );
        $self->push(
            new Sanger::Graphics::Glyph::Text({
                'x'         => -7,
                'y'         => (1 - $i / $maxy) * $scl ,
                'width'     => 3,
                'height'    => $Config->texthelper->height('Tiny'),
'font'      => 'Tiny',
'text'      => "$i",
                'absolutey' => 1,
                'absolutex' => 1,
            })
        );
    }


    #################################
    # Draw the one lod score drop down line
    #################################
    $self->push(
        new Sanger::Graphics::Glyph::Line({
            'x'         => 0,
            'y'         => (1 - ($max_score - 1)/$maxy) * $scl,
            'width'     => $chrlen,
            'height'    => 0,
            'colour'    => $clr[0],
            'absolutey' => 1,
    'dotted'    => 1,
        })
    );

    #################################
    # Draw the zoom position red box
    #################################
    my $zbox = new Sanger::Graphics::Glyph::Rect({
    'x'      => $slice->chr_start(),
    'y'      => 0,
    'width'  => $vclen,
    'height' => $scl,
    'bordercolour' => $clr[0],
'absolutey' => 1,
    });
    $self->push($zbox);


    #################################
    # Draw the x-axis
    #################################
    $self->push(
        new Sanger::Graphics::Glyph::Line({
            'x'         => 0,
            'y'         => $scl + 2,
            'width'     => $chrlen,
            'height'    => 0,
            'absolutey' => 1,
        })
    );
    my $maxx = int($chrlen / 10000000);
    for (my $i = 1; $i <= $maxx; $i++) {
        $self->push(
            new Sanger::Graphics::Glyph::Line({
                'x'         => $i * 10000000,
                'y'         => $scl + 2,
                'width'     => 1,
                'height'    => 3,
                'absolutey' => 1,
            })
        );
my $Mb = $i * 10;
        $self->push(
            new Sanger::Graphics::Glyph::Text({
                'x'         => $i * 10000000,
                'y'         => $scl + 6,
                'height'    => $Config->texthelper->height('Tiny'),
                'font'      => 'Tiny',
                'text'      => "$Mb",
                'absolutey' => 1,
            })
        );
    }
    $self->push(
        new Sanger::Graphics::Glyph::Text({
            'x'         => 270,
            'y'         => $scl + 20,
            'height'    => $Config->texthelper->height('Small'),
            'font'      => 'Small',
            'text'      => "Physical location (in Mb)",    
            'absolutey' => 1,
            'absolutex' => 1,
        })
    ); 

    #################################
    # Draw legend
    #################################
    $kc = 1;
    my ($X, $Y) = (0,0);
    my $new_scl = $scl + 40;
    my $LEG_S   = 60;
    my $BOX_H   = 8;
    my $BOX_W   = 20;
    my $COL_N   = 3;

    $self->push(
        new Sanger::Graphics::Glyph::Text({
            'x'         => 0,
            'y'         => $new_scl,
    'height'    => $Config->texthelper->height('Small'),
    'font'      => 'Small',
      'text'      => 'Legend',
    'absolutey' => 1,
    'absolutex' => 1,
        })
    );
    foreach my $key (sort keys %analysis) {
        $self->push(
            new Sanger::Graphics::Glyph::Line({
                'x'         => $LEG_S + ($im_width - $LEG_S - 10) * $X / $COL_N + 10,
'y'         => $new_scl + $Y * $BOX_H + 6,
'width'     => $BOX_W,
'height'    => 0,
'colour'    => $clr[$kc],
'absolutey' => 1,
'absolutex' => 1,
            })
         );
        $self->push(
            new Sanger::Graphics::Glyph::Text({
                'x'         => $LEG_S + ($im_width - $LEG_S - 10) * $X / $COL_N + $BOX_W + 12,
'y'         => $new_scl + $Y * $BOX_H + 4,
'height'    => $Config->texthelper->height('Tiny'),
'font'      => 'Tiny',
'text'      => $key,
'colour'    => $clr[$kc],
'absolutey' => 1,
'absolutex' => 1,
            })
         );
$kc++;
$X++;
if ($X == $COL_N) {
     $X = 0;
     $Y++;
         }
    }
}            
1;
