# Supplementary material for: Statistical Viewer: a tool to upload and integrate linkage and association data as plots displayed within the Ensembl genome browser
Source: BMC Bioinformatics. 2005 Apr 12;6:95. doi: 10.1186/1471-2105-6-95 (PMC1087836; doi:10.1186/1471-2105-6-95)
Supplement: Additional File 3 — The source code for Bio::EnsEMBL::GlyphSet::LodPlot [file 1471-2105-6-95-S3.rtf]

######################################################################
#                                                                    #
# Ensembl module for Bio::EnsEMBL::Linkage                           #
#                                                                    #
# Maintained by Hong Xu <hxu@chg.duhs.duke.edu>				   # 
# Center for Human Genetics Bioinformatics Core          		   #
# Duke University Medical Center                                     #
#                                                                    #
# You may distribute this module under the same terms as perl itself #
#                                                                    #
######################################################################


=head1 NAME

Bio::EnsEMBL::Linkage

=head1 SYNOPSIS

use Bio::EnsEMBL::Linkage;

# create and populate a linkage point
$lk = Bio::EnsEMBL::Linkage;
$lk->study('Gencard');
$lk->analysis('GCGS');
$lk->chr_name('1');
$lk->link_point('D1S1195');
$lk->start(72329855);
$lk->end(72329995);
$lk->score(3.618);


=head1 DESCRIPTION

Linkage objects encapsulate data pertaining to a single linkage point.
Access these objects through a Bio::EnsEMBL::DBSQL::LinkageAdaptor

=head1 AUTHOR

Hong Xu

This modules is part of the Ensembl project http://www.ensembl.org

=head1 CONTACT

Post questions to the EnsEMBL developer mailing list <ensembl-dev@ebi.ac.uk>  

=cut

package Bio::EnsEMBL::Linkage;

use strict;
use vars qw(@ISA);
use Bio::EnsEMBL::Root;
@ISA = qw(Bio::EnsEMBL::Root);


=head2 new

  Arg [1]    : none
  Example    : $lk = Bio::EnsEMBL::Linkage->new;
  Description: Constructor.  Creates a new Linkage object
  Returntype : Bio::EnsEMBL::Linkage
  Exceptions : none
  Caller     : Bio::EnsEMBL::LinkageAdaptor

=cut

sub new {
    my ($class) = @_;

    my $self = {};
    bless $self,$class;

    return $self;
}


=head2 study

  Arg [1]    : (optional) string $value
  Example    : my $study = $link->study(); 
  Description: Getter/Setter for the study name of this linkage
  Returntype : string
  Exceptions : none
  Caller     : general

=cut

sub study{
   my $self = shift;
   if( @_ ) {
      my $value = shift;
      $self->{'study'} = $value;
    }
    return $self->{'study'};
}


=head2 analysis

  Arg [1]    : (optional) string $value
  Example    : my $analysis = $link->analysis(); 
  Description: Getter/Setter for the analysis name of this linkage
  Returntype : string
  Exceptions : none
  Caller     : general

=cut

sub analysis{
   my $self = shift;
   if( @_ ) {
      my $value = shift;
      $self->{'analysis'} = $value;
    }
    return $self->{'analysis'};
}


=head2 chr_name

  Arg [1]    : (optional) string $chr_name
               Name of the chromosome this Linkage point is on 
  Example    : $chr_name = $link->chr_name; 
  Description: Getter/Setter for the name of the chromosome this linkage point is on 
  Returntype : string
               Name of the chromosom this linkage point is on
  Exceptions : none
  Caller     : general

=cut

sub chr_name {
   my $self = shift;
   if( @_ ) {
      my $value = shift;
      $self->{'_chr_name'} = $value;
    }
    return $self->{'_chr_name'};
}


=head2 link_point

  Arg [1]    : (optional) string $link_point
               Name of this Linkage point 
  Example    : $link_point = $link->link_point; 
  Description: Getter/Setter for the name of this linkage point 
  Returntype : string
               Name of this linkage point
  Exceptions : none
  Caller     : general

=cut

sub link_point {
   my $self = shift;
   if( @_ ) {
      my $value = shift;
      $self->{'link_point'} = $value;
    }
    return $self->{'link_point'};
}


=head2 start

  Arg [1]    : (optional) int $newvalue
  Example    : my $link_start = $link->start(); 
  Description: get/set for the linkage start (e.g. 10000) in absolute basepairs  
  Returntype : int 
  Exceptions : none
  Caller     : general

=cut

sub start{
   my $self = shift;
   if( @_ ) {
      my $value = shift;
      $self->{'start'} = $value;
    }
    return $self->{'start'};
}


=head2 end

  Arg [1]    : (optional) int $value
  Example    : $link_end = $link->end;
  Description: get/set for the linkage point end (e.g. 10000) in absolute basepairs  
  Returntype : int
  Exceptions : none
  Caller     : general

=cut

sub end{
   my $self = shift;
   if( @_ ) {
      my $value = shift;
      $self->{'end'} = $value;
    }
    return $self->{'end'};
}


=head2 score

  Arg [1]    : (optional) float $value
  Example    : my $score = $link->score(); 
  Description: get/set for the linkage score (e.g. 3.618) 
  Returntype : float 
  Exceptions : none
  Caller     : general

=cut

sub score{
   my $self = shift;
   if( @_ ) {
      my $value = shift;
      $self->{'score'} = $value;
    }
    return $self->{'score'};
}


1;
