# Supplementary material for: Statistical Viewer: a tool to upload and integrate linkage and association data as plots displayed within the Ensembl genome browser
Source: BMC Bioinformatics. 2005 Apr 12;6:95. doi: 10.1186/1471-2105-6-95 (PMC1087836; doi:10.1186/1471-2105-6-95)
Supplement: Additional File 4 — The source code for WebUserConfig::chrplot BioPerl module [file 1471-2105-6-95-S4.rtf]

######################################################################
#                                                                    #
# Ensembl module for WebUserConfig::chrplot	                               #
#                                                                    #
# Maintained by Hong Xu <hxu@chg.duhs.duke.edu>				   # 
# Center for Human Genetics Bioinformatics Core          		   #
# Duke University Medical Center                                     #
#                                                                    #
# You may distribute this module under the same terms as perl itself #
#                                                                    #
######################################################################


package WebUserConfig::chrplot;
use strict;
use WebUserConfig;
use vars qw(@ISA);
@ISA = qw(WebUserConfig);

sub init {
  my ($self) = @_;

  $self->{'_userdatatype_ID'} = 111;
  $self->{'_label'} = 'above';

  $self->{'general'}->{'chrplot'} = {
    '_artefacts' => [qw( lodplot )],
    '_options'  => [],
    '_settings' => {
      'width'   => 700,
      'show_contigview' => 'yes',
      'show_cytoview'   => 'yes',
      'bgcolor'   => 'background1',
      'bgcolour1' => 'background1',
      'bgcolour2' => 'background1',
    },
    'lodplot' => {
      'on'  => "on",
      'pos' => '1',
    }
    };
}
1;
