# Supplementary material for: Statistical Viewer: a tool to upload and integrate linkage and association data as plots displayed within the Ensembl genome browser
Source: BMC Bioinformatics. 2005 Apr 12;6:95. doi: 10.1186/1471-2105-6-95 (PMC1087836; doi:10.1186/1471-2105-6-95)
Supplement: Additional File 11 — Manual for using the Get Map tool for the interconversion of genomic and genetic positions for markers. The algorithm is also described. [file 1471-2105-6-95-S11.doc]

#
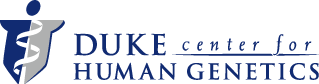


**Bioinformatics Core Resource**

**The CHG User’s Manual for Get Map:**

**A Web Tool for the Interconversion between Genomic Coordinates and Genetic Map Locations**

**Background**

- As we enter the post-genomic sequencing era, there is an increasing need for the interconversion between genome locations and genetic distances. In particular the integration of statistical data (e.g. linkage and association data) with human genome sequence browsers requires converting the genetic distance of markers on a chromosome (cM) to the genomic location in base pairs.
  - Examples of useful conversions include:
    - Gene location -> Gene genetic distance
    - SNP location -> SNP genetic distance
    - Multipoint genetic distance -> Genome location
    - Marshfield map -> deCODE map *
- The interconversion process is slow, tedious, and error-prone when performed manually
- Markers that map to several locations as well as those that exhibit inconsistencies in ordering can be problematic and can be avoided.

**Contents**

**Using Get Map**

**l. Accessing the Get Map Server** ………………………………………………...……..…….. **2**

**ll. Formatting the Input File**……………………………………………………………..……….. **3**

**lll. The Input Process** …………………………………………………………………..…...…..….. **3**

**lV. The Output**  ………………………………………………………………………….………..... **6**

**Materials and Methods**

**l. Constructing the Database of Marker Locations** …………….………….….…….. **7**

**ll. Implementation Strategy** ……………………………………………………...…….…..… **7**

**1. Preprocessing** …………………………………………..…………………..……......… **8**

**How Get Map Works**

**I. Modified Binary Search Algorithm** …..…………………………………………….…… **9**

**ll. Linear Interpolation Algorithm** …..…………………………………………….…..…… **10**

**References** ………………………………………………………………………….………..….…..…..... **12**

**Appendix** ……………………………………………………………………………………….………..... **13**

**Accessing the Get Map Server**

The Web Server is accessed through the Internal Ensembl Home Page:

http://genominator2.duhs.duke.edu. As private data pertaining to on-going studies is on the DAS server and is accessible through Ensembl, access is restricted. Therefore, the first time you access this you will need to enter the username and password and click the Okay button. By checking the box beneath the password you will not have to re-enter this information the next time you access this site.

**
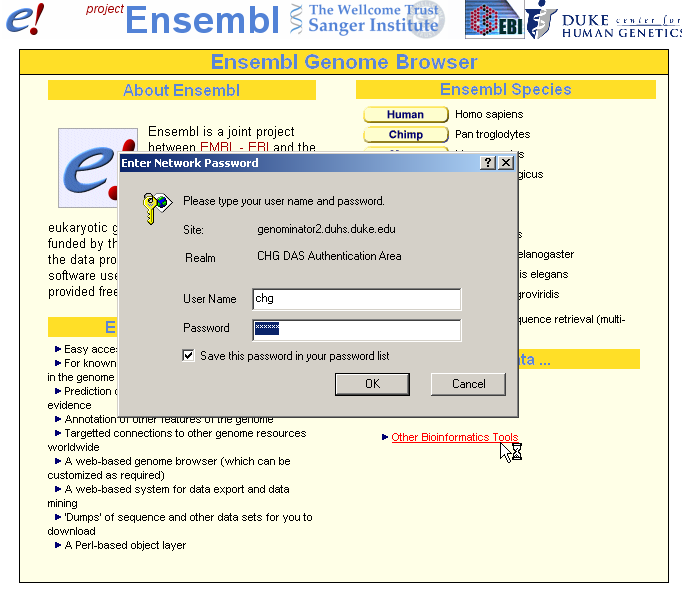
**

Fig. 1 The internal web page overlayed by the security window. The cursor is pointing to the link to the Get Gene and Get Map tools (in red text ).

Next click the link – “CHG DATA” under “CHG Data …” section on the lower right of the CHG Ensembl home page (visible in fig. 1 below). This will pop up a “Security alert” page (not shown). Click on the “Yes” button to proceed. This will bring up the page shown in Figure 3 in the section on “The Input Process”.

**Using GetMap**

### I. Formatting the Input Data

The first step is to put the data into the form of a file (either .txt or .xls) in which the:

1. marker name appears in the first column
2. chromosome identifier is placed in the 2^nd^ column
3. marker position (start coordinates) or genetic position is in the 3^rd^ column
4. in the case of converting from genomic location the end position of the marker in bps is placed in the 4^th^ column.

**Start Position**

**End Position**

**(bps)**

**Chromosome**

**Marker**

Figure2. An example of a correctly formatted (.xls) input file with known genomic coordinates (bps) provided

**II. The Input Process**

The GetMap web front-end can be accessed either through the CHG Ensembl home page (under CHG Data follow link to other bioinformatics tools) or by directly entering this URL:

<http://genominator2.duhs.duke.edu:8080/chg/tool.html>. From there the user can select among the six conversion options shown below with the necessary input fields specified:

1. **genome location -> deCODE/Genethon/Marshfield:** the Excel spreadsheet should have following fields: ID,Chr,Chr_start(bp),Chr_end(bp). (For an example see fig.1)
2. **deCODE -> genome location:** the input file should have following fields: ID, Chr, deCODE(cM).
3. **Genethon -> genome location**: Required input fields: ID, Chr, Genethon(cM).
4. **Marshfield -> genome location**:Required input fields: ID, Chr, Marshfield(cM).
5. **Marshfield -> deCODE**: Required input fields: ID, Chr, Marshfield(cM).
6. **Genethon -> deCODE**: Required input fields: ID,Chr,Genethon(cM).

**
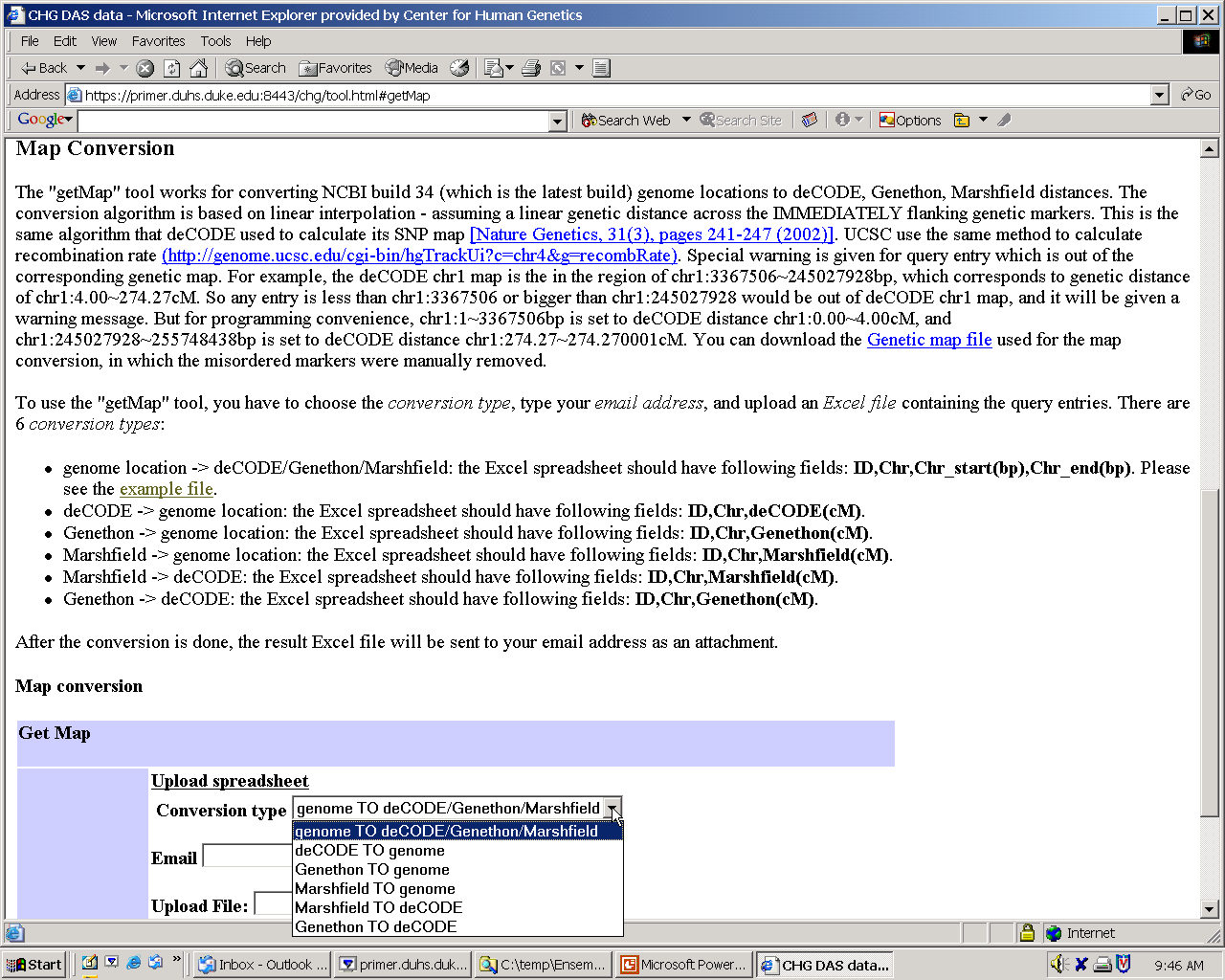
**

Figure 3. Web-based server form page for uploading data for position conversion. Note: another tool called GetGene that will return a list of genes within a specified region is at the top of this “CHG Bioinformatic Tool” page.

Next, the necessary information for the following for the 3 remaining fields must be supplied as illustrated in figure 4:

- 1. Your email address
  2. The path specifying the location of the input file must be chosen using the browse tool
  3. The format of the input file must be selected from the pull down menu as GetMap can also accept tab-delimited text files (supplying the necessary fields) as input

Finally click on the upload button.

**
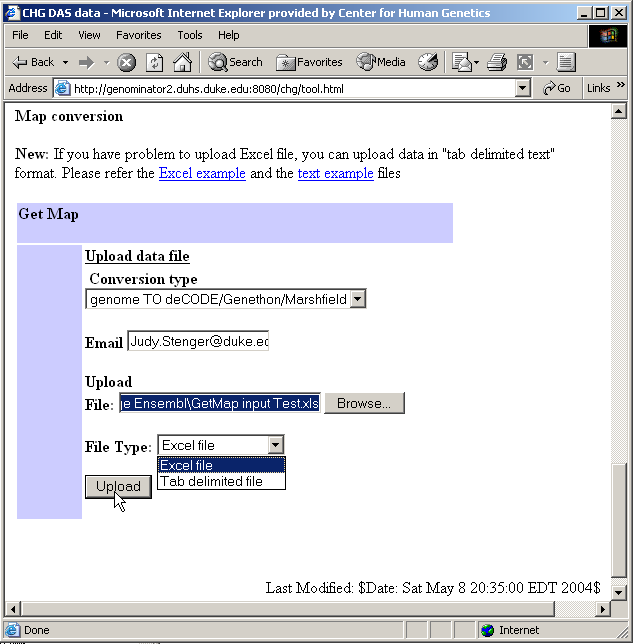
**

Fig 4. Web-based server form page for uploading data for position conversion illustrating the browse tool for finding the path of the upload file, the file type pull-down menu and the upload button

Once the data has been submitted to the server. The web page then changes to indicate “The conversion results will be sent to your email


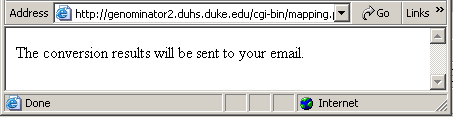


Figure 5. The web page displayed after the input data has been submitted

**III. The Output**

- An email is returned to the address supplied by the user with the results contained in an attached Excel file such as that shown below in figure 2.

**
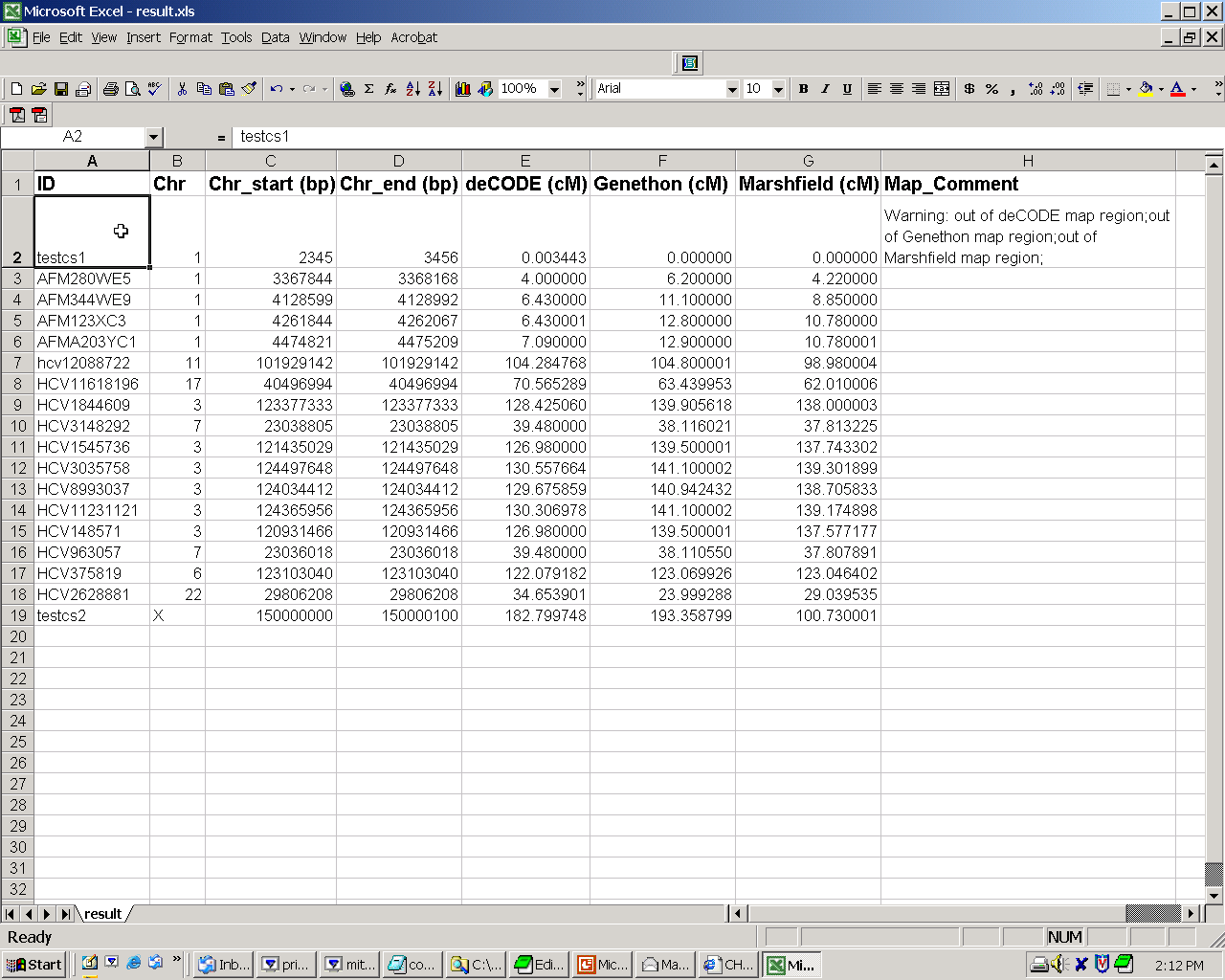
**

Figure 6. An example of the output returned by GetMap after processing the input data shown in figure 1. Note that the computation output is carried out to the 1X10^-6^ position during the calculations, although the data should not regarded with this level of precision that this may imply

**Materials and Methods**

### I. Constructing the Database of Marker Locations

Figure 7. Flow chart illustrating how GetMap works. First all markers with their location data are downloaded from the three major genetic databases. The deCODE genetic map^6^ ,Marshfield^7^ , and Généthon8 genetic maps are the primary sources for the genetic locations. The marker sequences and genetics positions are obtained from dbSTS (NCBI). The genomic locations are mapped by e-PCR to the most recent Human Genome Sequence Assembly (NCBI HG build #34 is obtained from UCSC d e-PCR

**Database** (of locations for Unique Markers with consistent ordering)

**Généthon**

**Marshfield**

**deCODE**

**Genomic**

**ID**

**Web Front-End Module**

Parses

input file

Generates results

e-mails output to

user

**Middle Layer**

Modified Binary Search

Returns location for query markers; else

Calculate best estimated location

Finds adjacent flanking markers and returns their positions

Uses flanking positions as input parameters for linear interpolation

**Preprocessing**

Data is smoothed by removing markers that map to several regions of the genome or that are inconsistently ordered

**Download Public Data**

Marker locations for

Genetic Maps

Human Genome

Assembly (current

Build: HG #34)

Marker Sequences (STS)

**Map to Genome**

__________________________________________

### II. Implementation Strategy

- **Retrieve Marker Data:** Download UniSTS data and the deCODE Marshfield, Marshfield and Généthon genetic maps from NCBI FTP server ([ftp.ncbi.nlm. nih.gov/repository/UniSTS/](ftp://ftp.ncbi.nlm.nih.gov/repository/UniSTS/))
- **Retrieve Genome Assembly:** Download human genome sequence data from UCSC server (<http://genome.ucsc.edu/goldenPath/hg16/bigZips>).
- **Find Genomic Locations:** Use e-PCR [5] or BLAT[6] to map STS markers on human genome assembly (NCBI 34).
- **Data Smoothing:** Check map results for duplicated, mis-ordered (inconsistent) or mismatched markers. These markers are removed and the remaining pre-processed markers are loaded to MySql database.

1. **Preprocessing (Data Smoothing)**

Once the microsattelite markers are mapped, results are screened to identify inconsistancies that we refer to as duplicated, misordered or mismatched (See fig. 8). These abnormal markers are removed. We generate a slope for the markers at the same genetic distance

**2) Remove mis-ordered markers**

**1) Remove markers that are map to more than 1 location on a Chromosome**

Remove inconsistently ordered markers

Remove markers with “mismatched” chromosomes

**3) Remove markers with inconsistent positional data**

Figure 8. Illustration of the data smoothing performed during preprocessing in constructing GetMap’s database of filtered marker IDs and their locations. The tables with the yellow background are prior to data smoothing. Red arrows and red text denote data that is problematic in ordering and mapping and thus should not be included since they cause confounding results. The tables with the blue background show “smoothed data”.

é

**How GetMap Works**

The GetMap program essentially uses modified versions of two well-known algorithms;

The binary search and linear interpolation.

**I. Modified Binary Search Algorithm**

The binary search is much more efficient than a linear search as illustrated by the data in table 1. This algorithm employs search trees[9] to locate a key by performing the operation find(*k*) on the MySQL ordered database of unique markers. This database can be conceptualized as array-based sequences of records that are ordered according to a key (e.g. location).

- The features of the algorithm are:

1. At each step, the number of candidate items is halved.
2. After O(log n) steps, the algorithm terminates, substantially reducing the number of steps.

Table 1. Advantages of the binary search over a linear search

For example, see the binary search of an ordered array of integers of length 13 illustrated in figure 9. int A[13], is initialized with the values ( 0, 1, 3,4, 5, 7, 8, 9, 11, 14, 16, 18, and 19) in positions A[*i* ] where *i* = 0; *i* < 13, *i*++. Therefore, to find the location holding the value 7 in the ordered array, A, it take log2N steps, where N =13. Note that in step 4, The positions low (l), middle and high (h) converge ( *l = m = h* ) at A[5], the location holding the value 7.

**[0] [1] [2] [3] [4] [5] [6] [7] [8] [9] [10] [11] [12]**

**Step**

**1) A[*i*]**

**2) A[*j*]**

**3) A[*k*]**

**4)**


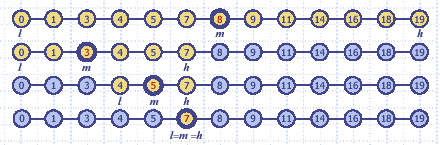


Figure 9. Diagram of different binary searches paths taken to find the index containing the value 7. Each row represents a step. The columns represent the indicies [*i* ], where *i* = {0 - 12) for array, A. The values are ordered. The middle of the linear array is first chosen, essentially cutting the array in half. As the list is ordered, since the middle value *( m)*  contains a value > 7 , the 2^nd^ half of the array is excluded in step B. This time the 3^rd^ column is selected as the middle (*m*). Since A[2] = 3. Since 3 < 7, the first half of the array (now A[ *j* ], where *j* = {0-5}) is excluded from step 3, leaving A[*k* ] where *k* = {3 – 5}. As the search space is now only three positions, the next step will choose A[4] as the middle, guaranteeing that the value will be located in the next step.

**II. Linear Interpolation**

An example problem to which GetMap may be applied is provided below:

*Suppose we know the genomic position of the SNP rs1329853 and would like to include to incorporate this into a genetic map that we have been using based upon our linkage analysis for a particular study, but we need a good approximation of the deCODE location (sex averaged) to place this marker on our map.*

To obtain an approximate genetic location (cM) for the SNP rs1329853 (with respect to the deCODE map) GetMap first employs a modified binary search of the MySQL db of legitimate markers to find the identifier and the databased genetic position of the query. As a SNP rs1329853 is not currently in the database, the binary search function of GetMap selects the

nearest flanking markers with both deCODE and genomic positions, D9S1870 & D9S171, and returns their positions to use as input parameters for the interpolation algorithm.

To calculate the approximal genetic distance of a marker, we assume there is a linear genetic distance across the closest adjacent flanking genetic markers in the pre-processed database

For this example the genetic location of rs1329853 is calculated as follows:

1. Determine the ratio of base pairs per centimorgan between the two nearest unique flanking markers that are consistently ordered

Dist. Between flanking marker(cMs)

Dist. Between flanking marker(bps)

**(bps)**

**Dist. betw flank markers (bps)**

45.57 – 43.44 cMs

24524318-22093115 bps

**(bps)**

**Dist. betw flank markers (bps)**

2.13 cMs

2431203 bps

**(bps)**

**Dist. betw flank markers (bps)**

Ratio = ­­­­ = = = 8.76 X 10-7 cM/bp

1. Determine the distance in (bps) between the query marker and the left flanking marker:

Distance (bps) = Query Position - Left adjacent Flank Position

= 24,518,892 *(rs1329853*) – 22,093,115 (*D9S1870*)

= 2,425,777 bps

1. Estimate the genetic distance between the query and the left flanking marker by multiplying the ratio is multiplied by the distance in between the query and left flanking marker

Estimated genetic distance (*between query and left flank*) = Ratio (cM/bp) X Distance (bps)

= (8.76 X 10-7) cM/bp X 2,425,777 bps

= 2.125

4. Get the estimated genetic location of the query by adding the estimated genetic distance (in cM) determined in step 3 to the genetic location of the left flanking marker.

Rs1329853 (cM) = left flank genetic position + estimated genetic distance= 45.56 cM

= 43.44 cMs + 2.125 = 45.56

Table 2. The estimated deCODE position for SNP rs1329853 as calculated by Get Map. The requested output is returned to the user in an excel file.

**References**

1. Deloukas, P., et al. (1998). A physical map of 30,000 human genes. *Science*. 282:744-746.
2. Rosen, N., et al. (2003) GeneLoc: Exon-based integration of human genome maps. *Bioinformatics* 19(S1):i222-i224.
3. Kong, A., et al. (2002). A high-resolution recombination map of the human genome. *Nature Genetics*. 31(3):241-247.
4. Schuler, GD. (1997) Sequence mapping by electronic PCR. *Genome Res.* 7:541-550.
5. Kent, J. (2002) BLAT - The BLAST-Like Alignment Tool. Genome Res. 12:656-664.
6. Kong, A. et al. Nat Genet. 2002 July; 31(3): 241-7.
7. Broman, K.W. et al. Am. J. Hum. Genet. 1998; 63:861-869
8. Cohen, D et al. Nature 1993 December; 336(6456):698-701.
9. Goodrich, M.T., Tamassia, R. and Mount, D.M. “Chapter 9: Search Trees” *in* Data Structures and Algorithms in C++. John Wiley & Sons, Inc. New York. 2003. <http://cpp.datastructures.net/presentations/BinarySearchTrees.pdf>

**Appendix**

**2004 CSHL Genome Meeting Abstract**

**GetMap:**

**A Web Tool for the Interconversion between Genomic Coordinates and Genetic Map Locations**

**Hong Xu, Elizabeth Hauser and Judith E. Stenger**

Center for Human Genetics, Duke University Medical Center, P.O. Box 3445, Durham, North Carolina 27710, USA.

**Abstract**

Since the completion of the first human genome draft, more researchers are using an integrated approach towards identifying and prioritizing candidate disease susceptibility genes. With such an approach there is a need to integrate genomic and genetic data with other research data. To facilitate the integration, marker locations must be easily converted from genetic positions (mapped in centimorgans) and genome assembly coordinates (denoted by base pairs) to the other data unit, or vice versa. Although some applications were developed to address this problem, they were either limited to gene features [1,2] or based on out-dated genome working draft [3].

Here we describe a web tool developed to facilitate the interconversion of marker positions between various genetic map distances (e.g. deCODE, Marshfield, or Généthon) and the bp coordinates of the most recent human genome sequence assembly release. First, microsatellite markers of deCODE, Marshfield, and Généthon are mapped to NCBI human genome build 34 using e-PCR [4] or BLAT [5]. Markers with mismatched genomic order and genetic order are removed from the marker lists. Then the filtered markers (98.23% of deCODE markers, 83.08% of Marshfield markers, and 82.14% of Généthon markers) are put into a MySql database. The web front end uploads text or Excel files provided by the user. The algorithm parses the file and finds the immediately flanking genetic markers for each query point. When the conversion is from genome location to genetic distance, genetic distance is calculated by linear interpolation, assuming a linear genetic distance across the immediately flanking genetic markers. When the conversion is from the genetic distance to genome location, the genome location is searched by marker name first. If no match is found, genome location is also calculated by linear interpolation. Finally the web tool sends the output results to the user as an Excel file attached to the email. A standalone version of the web tool is developed for running batch conversion, such as converting large number of SNP locations to genetic distances.
